# Eupatilin attenuates the senescence of nucleus pulposus cells and mitigates intervertebral disc degeneration *via* inhibition of the MAPK/NF-κB signaling pathway

**DOI:** 10.3389/fphar.2022.940475

**Published:** 2022-11-03

**Authors:** Huan Yang, Xiao Yang, Kewei Rong, Jiarong Liang, Zhengting Wang, Jie Zhao, Pu Zhang, Yijie Li, Lihuan Wang, Hui Ma, Bin Ye

**Affiliations:** ^1^ Shanghai Key Laboratory of Orthopedic Implants, Shanghai Ninth People’s Hospital, Shanghai, China; ^2^ Second Clinical Medical College, Yunnan University of Traditional Chinese Medicine, Kunming, Yunnan, China; ^3^ Department of Orthopedics, Ninth People’s Hospital, Shanghai Jiaotong University School of Medicine, Shanghai, China; ^4^ Yunnan College of Business Management, Kunming, Yunnan, China; ^5^ South Branch of Zhaotong First People’s Hospital, Zhaotong, Yunnan, China; ^6^ Northeast Yunnan Regional Central Hospital, Kunming, Yunnan, China; ^7^ Yunnan St. John’s Hospital, Kunming, Yunnan, China

**Keywords:** Eupatilin, intervertebral disc degeneration, inflammation, senescence, extracellular matrix, nucleus pulposus cells

## Abstract

Intervertebral disc degeneration (IDD) is the main cause of low back pain. An increasing number of studies have suggested that inflammatory response or the senescence of nucleus pulposus (NP) cells is strongly associated with the progress of IDD. Eupatilin, the main flavonoid extracted from *Artemisia*, was reported to be associated with the inhibition of the intracellular inflammatory response and the senescence of cells. However, the relationship between eupatilin and IDD is still unknown. In this study, we explored the role of eupatilin in tumor necrosis factor-α (TNF-α)-induced activation of inflammatory signaling pathways and NP cell senescence, in the anabolism and catabolism of NP cell extracellular matrix (ECM) and in the effect of the puncture-induced model of caudal IDD in the rat. *In vitro*, eupatilin significantly inhibited TNF-α-induced ECM degradation, downregulated the expression of related markers of NP cells (MMP3, MMP9, and MMP13), and upregulated the expression of *SOX9* and *COL2A1*. Furthermore, eupatilin reduced TNF-α-induced cell senescence by inhibiting the expression of the senescence of NP cell-related markers (p21 and p53). Mechanistically, ECM degradation and cell senescence were reduced by eupatilin, which inhibited the activation of MAPK/NF-κB signaling pathways. Consistent with the *in vitro* data, eupatilin administration ameliorated the puncture-induced model of caudal IDD in the rat. In conclusion, eupatilin can inhibit the inflammatory response and the senescence of NP cells, which may be a novel treatment strategy for IDD.

## Introduction

With the extension of the human life span, the prevalence and impact of age-related diseases are also increasing. In a recent global survey of chronic diseases, low back pain (LBP) ranked first in the number of years of disability (GBD, 2018). Although the etiology of LBP is multifactorial, intervertebral disc degeneration (IDD) is the main cause of LBP (Risbud and Shapiro, 2014). Importantly, cell inflammation and senescence can exacerbate the progression of IDD. Therefore, there is an urgent need to understand the potential mechanisms of intervertebral disc inflammation and senescence and develop solutions to delay or improve the progression of IDD (Rea et al., 2018; Li et al., 2021).

The intervertebral disc (IVD) is flexible and plays a key role in regulating the biomechanical load acting on the spine. IVD is composed of the central gel nucleus pulposus (NP) tissue, annular ligament fibrous ring, and cartilage endplate. NP cells play a central role in the secretion of extracellular matrix (ECM) proteins, especially including aggrecan and type II collagen (Fiordalisi et al., 2020; Luo et al., 2021). IDD is closely related to the synthesis and catabolism of ECM of NP cells, including the decrease of the abundance and quality of the ECM, the increase of inflammatory mediators and catabolic processes, and the changes in the cell phenotype and senescence (Choi et al., 2018). Despite the widespread prevalence and huge economic burden, there is no effective treatment for IDD.

An increasing number of studies have shown that the IVD onset has been shown to have a genetic etiology or to be a consequence of trauma, or senescence and inflammation of NP cells. As a consequence of one of these factors or multiple factors, there is an unbalance in the catabolism/anabolism activity of NP cells that results in less production of the healthy matrix, degradation of the existing matrix, and the synthesis of inflammatory precursors (Patil et al., 2019; Wang et al., 2020). Among them, inflammatory and senescence of cells lead to upregulation of catabolism of NP cells, gradual reduction of type II collagen (COL2A1), and increase of matrix metalloproteinase proteins (MMPs), which results in an imbalance of ECM metabolism of NP cells and eventually ECM degradation (Zhang et al., 2020). These changes reduce the osmotic pressure in the intervertebral disc, the dehydration of the intervertebral disc matrix occurs, the outflow of matrix molecules increases, and many cytokines influx (IL-1β and TNF-α). At the same time, the inflammatory factors secreted by NP cells also increase (Zhao et al., 2020). With the degradation of the ECM, NP cells gradually decreased and were replaced by fibrotic tissues. This leads to the formation of annular fissures and the reduction of the disc height, which in turn leads to spinal stenosis, LBP, and nerve compression (Cherif et al., 2020). TNF-α is a key inflammatory factor that accelerates the senescence of NP cells and induces the expression of inflammatory mediators (Khan et al., 2017). It is also upregulated in the progression of IDD, including enhancing the inflammatory response, stimulating ECM degradation, and accelerating cell senescence (Liu et al., 2015; Li et al., 2017b; Yang et al., 2019). In short, TNF-α stimulates NP cells, the expression of MMPs increases, and COL2A1 decreases, eventually leading to the loss of ECM and the development of IDD (Li et al., 2018). Therefore, it can effectively suppress TNF-α-induced NP cell senescence, and inflammatory mediators may treat IDD.

TNF-α activates TNF-R to induce the upregulation of inflammatory cytokines and leads to DNA damage (Sedger and McDermott, 2014). Mitogen-activated protein kinase (MAPK) and nuclear factor-kappa B (NF-κB) pathways are considered the main inflammatory responses to TNF-α stimulation in NP cells (Wang et al., 2018a; Huang et al., 2018; Huang et al., 2020). TNF-R is activated by TNF-α to induce upregulation of inflammatory cytokines to induce NF-κB release into the nucleus and trigger the transcription of many genes (Li et al., 2017c; Huang et al., 2018). In addition, TNF-R can also activate P38 MAPK and cause DNA damage (Li et al., 2017c). These changes lead to an increase in double-stranded DNA (dsDNA) in the cytoplasm of NP cells, leading to cell senescence and abnormal inflammatory factor infiltration (Guo et al., 2019). These signaling pathways cause a vicious cycle of the inflammatory cascade and accelerate the development of IDD. This suggests that inhibiting the inflammatory factor pathway or improving the inflammatory response in time may delay the progress of IDD. Eupatilin, the main flavonoid extracted from *Artemisia*, was reported to be associated with the inhibition of intracellular inflammatory response and the senescence of cells (Nageen et al., 2020).

Previous studies have reported that eupatilin inhibits NF-κB and MAPK signaling pathways significantly attenuating allergic inflammatory response (Song et al., 2018), reduces ova-induced asthma (Bai et al., 2022), and inhibits TNF-α-induced MMP expression (Jung et al., 2017). Furthermore, through the NF-κB signaling pathway, it inhibits oxidative damage and promotes the production of ECM in chondrocytes (Jeong et al., 2015). In this study, we conducted an experiment based on TNF-α-induced NP cell inflammation and studied the anti-inflammatory and anti-senescence effects of eupatilin. We also explored the potential mechanisms of these effects. In addition, our *in vivo* experiments of the puncture-induced model of caudal IDD in rats confirm the therapeutic effectiveness of eupatilin for IDD.

## Materials and methods

### Nucleus pulposus cell line and culture

The NP cell lines of Sprague–Dawley (SD) rats were provided by Dr. Chen Di from the Department of Orthopedic Surgery, Rush University Medical Center (Chicago, IL, United States). The cells were cultured in Dulbecco’s modified Eagle medium (DMEM) containing 5% fetal bovine serum (FBS) and 1% penicillin–streptomycin (GIBCO, Thermo Fisher Scientific, Waltham, Ma, United States).

### Nucleus pulposus primary cell isolation and culture

Five-week-old male SD rats (Shanghai Laboratory of Shanghai Animal Research Center, China) were killed by cervical dislocation and soaked in 75% ethanol for 10 min. NP tissues were obtained from the caudal intervertebral disc (Co1—Co8) through aseptic operation (Supplementary Figures S1C,D) and then digested in a CO_2_ incubator containing 0.25% type II collagenase for 2 h. After centrifugation (3000r/min) for 5 min, the supernatant was discarded. Then, the cells were resuspended in DMEM with 15% FBS and 1% penicillin–streptomycin (Gibco, Thermo Fisher Scientific, Waltham, MA, United States). All cells were cultured at 37°C, 21% O_2,_ and 5% CO_2_. The CDH2 marker genes of NP primary cells were identified during the subculture (Supplementary Figure S1E).

### Reagents and preparations

For *in vivo* and *in vitro* experimental studies, eupatilin (purity ≥ 98.8%) was purchased from Selleck Chemicals (Article No. 3,846, Shanghai Lanmu Chemical Co., Ltd.). Eupatilin was dissolved in DMSO as a 100 mM stock solution and stored at −80°C. To reduce cytotoxicity, the final concentration of DMSO was less than 0.1%.

### Cell counting Kit-8 assay cytotoxicity and proliferation

NP cells' toxicity and proliferation were detected using the CCK-8 kit (Sangon Biotech Co., Ltd., Shanghai, China). To determine toxicity, NP cells seeded in 96-well plates at a density of 2 × 10^4^ cells per well were cultured for 24 h with eupatilin at concentrations of 0, 1.56, 3.12, 6.25, 12.5, 25, 50, or 100 μM. To determine proliferation, cells seeded in 96-well plates at a density of 2 × 10^3^ cells per well were cultured for 24, 48, 72, and 96 h with eupatilin at concentrations of 0, 1.56, 3.12, 6.25, 12.5, 25, 50, or 100 μM. At the end of each experimental period, the medium was replaced with 10 µl of CCK-8 reagent and 90 µl of complete DMEM for 2 h in a 37°C incubator. Then, the optical density values of each well were recorded on a spectrophotometer at 450 nm on an Infinite M200 Pro Multimode Microplate Reader (Tecan Life Sciences, Männedorf, Switzerland).

### High-density culture and Alcian blue staining

To evaluate cell differentiation, 15 × 10^4^ NP primary cells were resuspended in a 10-μl medium and seeded at the bottom of a 24-well plate. The cells were allowed to adhere at 37°C for 1 h; then, 1 ml of DMEM containing 10 ng/ml ITS and 2% FBS was added. The medium was changed every other day. After 9 days, the Alcian blue stain was used for NP primary cell differentiation assay.

### Senescence assays

According to the manufacturer’s instructions, senescent NP primary cells were measured using the senescence β-galactosidase staining kit (Cell Signaling Technology, Danvers, MA, United States). In other words, NP cells were treated with 20 ng/ml of TNF-α and treated with or without eupatilin (6.25 μM, 12.5 μM) for 3 days. After 3 days of culture, the cells were fixed with 1x fixing solution at room temperature for 15 min and then were incubated at 37°C overnight without CO_2_ with the senescence β-galactosidase staining solution for 24 h. The images were taken under a bright field microscope (Olympus CKX41, Jan). These SA-β-Gal–positive cells were measured by ImageJ software (National Institutes of Health, United States).

### Total RNA isolation and real-time quantitative PCR analyses

According to the instructions, the TRIzol reagent (Thermo Fisher Scientific, Waltham, MA, United States) was used to isolate total RNA from tissues and cells. The first strand complementary DNA (cDNA) was reverse transcribed from the extracted RNA by the cDNA Synthesis Kit (TaKaRa Bio, Otsu, Japan). The TB Green Premix Ex Taq Kit (TaKaRa Bio) was used to perform real-time qPCR on the Applied Biosystems Quantum Studio 6 Flex Real-Time PCR System (Thermo Fisher Scientific). Specific primer pairs were designed using NCBI BLAST and the sequences provided in Table 1. Relative target gene expression was normalized to the CT value of GAPDH using the 2^−ΔΔCt^ method.

**TABLE 1:** Primer information.

Gene	Primer sequence	5′-primer-3′
*MMP3*	Forward	CCT​CTG​AGT​CTT​TTC​ATG​GAG​GG
	Reverse	ACT​TGA​GGT​TGA​CTG​GTG​CC
*MMP7*	Forward	CAA​AGG​ACG​ACA​TTG​CAG​GC
	Reverse	ATT​GCT​GGT​GTC​TGT​CGT​GT
*MMP9*	Forward	GAT​CCC​CAG​AGC​GTT​ACT​CG
	Reverse	GTT​GTG​GAA​ACT​CAC​ACG​CC
*MMP13*	Forward	ACC​ATC​CTG​TGA​CTC​TTG​CG
	Reverse	TTC​ACC​CAC​ATC​AGG​CAC​TC
*SOX9*	Forward	TCC​CCG​CAA​CAG​ATC​TCC​TA
	Reverse	AGC​TGT​GTG​TAG​ACG​GGT​TG
*TNF-α*	Forward	GCA​TGA​TCC​GAG​ATG​TGG​AAC​TGG
	Reverse	CGC​CAC​GAG​CAG​GAA​TGA​GAA​G
*IL-1β*	Forward	CAG​AAC​ATA​AGC​CAA​CAA​GT
	Reverse	ACA​CAG​GAC​AGG​TAT​AGA​TTC
*GAPDH*	Forward	GCA​TCT​TCT​TGT​GCA​GTG​CC
	Reverse	GAT​GGT​GAT​GGG​TTT​CCC​GT

### Protein extraction and Western blot analyses

Total cell proteins were extracted from cultured cells using RIPA lysis buffer (Roche, Basel, Switzerland) with phosphatase and protease inhibitors. The extracted protein was separated by equal weight (20–25 g) in the 4%–20% SurePAGE™ gel and electroblotted onto a 0.22-m PVDF membrane (Merck Millipore). The membrane was blocked with 5% BSA-TBST at room temperature for 1 h and then incubated with a primary antibody (diluted 1:1000 in 5% BSA-PBS) at 4°C overnight (at least 14 h). Primary antibodies including SAPK/JNK (#9252), IκBα (#4814), p-IκBα(#2859), phospho-SAPK/JNK (#4668), p65 (#8242), p38 MAPK (#54470), phospho-p38 MAPK (#4511), phospho-p44/42 MAPK (#4370), p44/42 MAPK (#4695), p-p65 (#3033), and β-actin (#8457) were purchased from Cell Signaling Technology (Denver, MA, United States). Additional primary antibodies including Anti-Col2a1 (ab34712), Anti-SOX9 (ab185966), Anti-MMP9 (ab58803), Anti-MMP13 (ab39012), Anti-p21 (ab109199), and Anti-p53 (ab26) were purchased from Abcam (Cambridge, United Kingdom). Secondary antibodies including anti-rabbit and anti-mouse antibodies were purchased from Cell Signaling Technology. On the second day, the membranes were washed in Tris-buffered saline Tween 20 (TBST) and cultured with anti-rabbit IgG (H + L) (DyLight) ™ secondary antibody (1:5000 dilution) at room temperature for 1 h in the dark. Then, the membranes were extensively washed in TBST, and the protein immunoreactivity was detected on the Li-Cor Odyssey Fluorescence Imaging System (Li-Cor Biosciences, Lincoln, northeast, United States). The intensity of protein immunoreactive bands was measured by ImageJ software using β-actin as an internal reference.

### Animals and surgical procedures

All surgical procedures were approved by the Institutional Animal Care and Ethics Committee of Ninth People’s Hospital, Shanghai Jiaotong University School of Medicine (Shanghai, China). Six 8-week-old male Sprague–Dawley rats were exposed to 26°C–28°C and 50%–65% humidity for 12 h day and night. The animals were fed standard rodent food and had free access to fresh water. They were fed standard rodent food and had free access to fresh water. Before the operation, the rats were anesthetized by an intraperitoneal injection of pentobarbital sodium (5 mg/100 g body weight). After tail disinfection, a longitudinal skin incision was made on the ventral side of the tail to expose 6–10 IVD. The Co6/7 IVD was used as the sham control, and Co7/8, Co8/9, and Co9/10 were used as the experimental groups. The IVD was used to perform a transverse puncture (10 mm) with a 20-caliber sterile needle perpendicular to the skin, rotated 180°, and remained for 10 s. PBS (10 μl) was injected into the Co7/8 group and eupatilin into the Co8/9 (12.5 μM, 10 μl) and the Co9/10 (25 μM, 10 μl) groups. The same method was used again 2 weeks after the operation. Four weeks after the operation, all rats were killed by cervical dislocation, and the caudal vertebrae were examined by MRI and X-ray. The samples were fixed in 4% PFA for subsequent downstream analyses.

### Radiographic analysis

The tails of all rats were examined by X-ray and MRI. The intervertebral disc height index was measured and calculated by the RadiAnt DICOM Viewer. Disc height index (DHI) formula: DHI = IVD height/adjacent IVD body height. Then, the DHI ratio was calculated to evaluate the height change of the intervertebral disc. The degeneration of the rat caudal intervertebral disc was observed on the MRI T2 image.

### Histology and immunofluorescence staining

The fixed IVD tissue was decalcified, dehydrated, and embedded in paraffin. These samples were then sectioned (4 μm thickness). For histological evaluation, paraffin tissue sections were stained with safranin O-Fast green and hematoxylin and eosin staining, according to standard laboratory procedures.

For cellular immunofluorescence, NP cells were fixed with 4% paraformaldehyde and permeabilized with 0.25% Triton X-100. The slides were washed three times with PBS and blocked with a blocking buffer for 30 min at room temperature. Then, the slides were incubated with primary antibodies overnight at 4°C. The primary antibodies used were p-p65 (Servicebio, 1:200), MMP3 (Servicebio, 1:200), and COL2A1 (Servicebio, 1:200). For tissue sections, they were washed three times with PBS and cultured in the antigen recovery buffer (Roche) at 37°C for 30 min. An autofluorescence Quencher was added to the slices for 5 min and blocked with a blocking buffer for 30 min at room temperature. Then, the sections were incubated with the primary antibody COL2A1 (Servicebio, 1:200) in a wet box overnight at 4°C. The next day, the slides were washed with PBS and treated with a secondary antibody (Servicebio, 1:500) in the dark at room temperature for 50 min. Subsequently, the slides were washed with PBS and cultured in the dark with DAPI (Servicebio, China) solution under RT for 10 min to stain the nuclei. The slices were washed with PBS, dried, and sealed with an anti-fluorescence quencher. The digital fluorescence image was taken under a Leica DM4000 B apparent fluorescence microscope (Leica Microsystems), and the IOD was measured by Image-Pro Plus 6.0 software.

### Statistical analysis

The results were analyzed by GraphPad Prism 9.0 (GraphPad Software Inc., San Diego, CA, United States). The data were presented as means ± standard deviation (SD). The comparisons between groups were made by one-way ANOVA. The statistically significant difference was set at *p < 0.05.

## Results

### Effects of eupatilin on cytotoxicity and cell proliferation of nucleus pulposus cells

**FIGURE 1:**
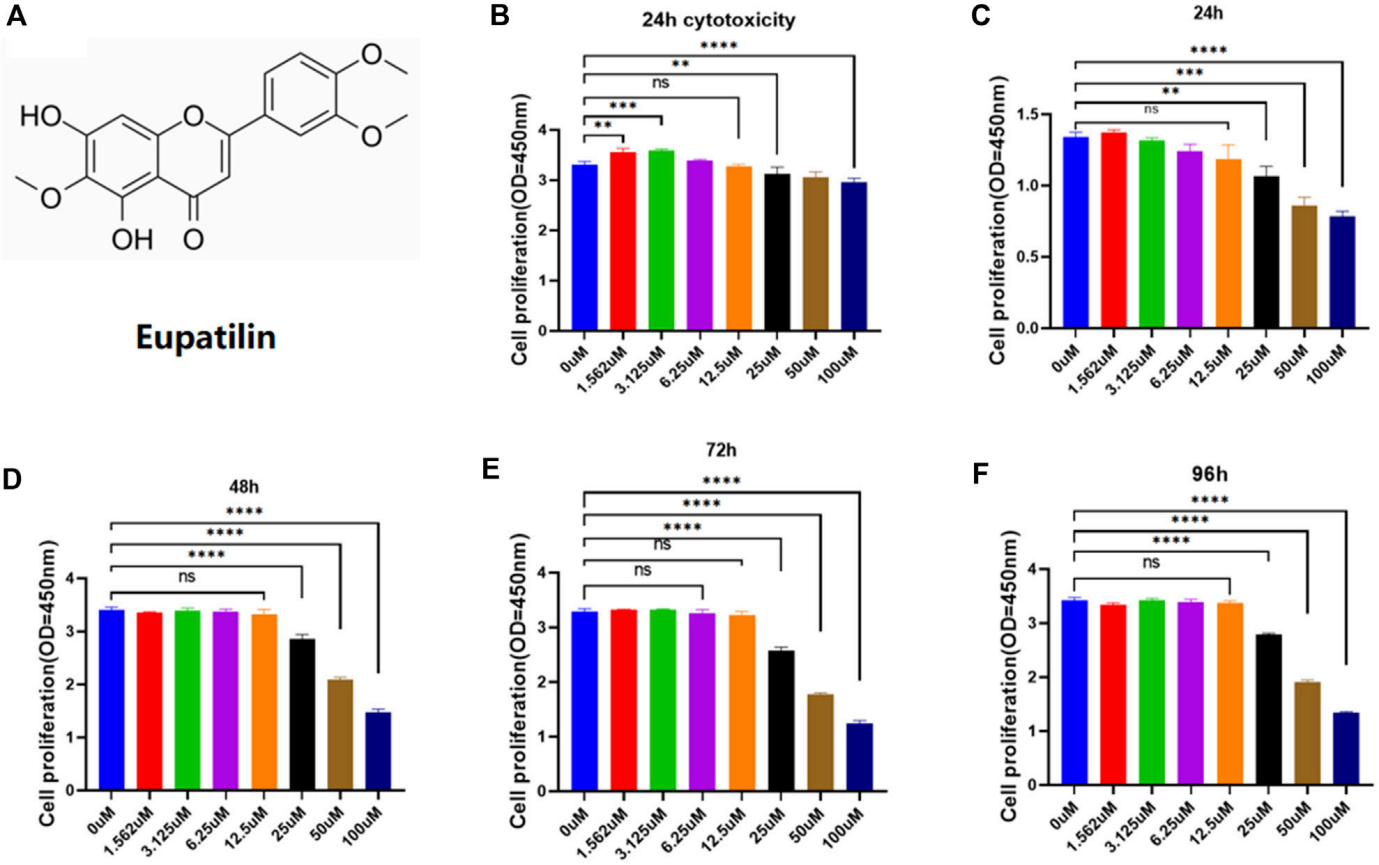
Eupatilin effects on proliferation and cytotoxicity of NP cells. **(A)** Chemical structure of eupatilin. **(B)** Effect of 24-h eupatilin 100 µM on NP cytotoxicity was determined by CCK-8. The 96-well plates contain 10,000 NP cells per well (*n* = 4). **(C–F)** Effects of eupatilin on the proliferation of NP cells in 24, 48, 72, and 96 h in the concentration range of 100 µM were determined by CCK-8 assay. The 96-well plates contain 2,000 NP cells per well. All the aforementioned data are expressed as mean ± SD. *n* = 4, compared with the control group. *p < 0.05, **p < 0.01, ***p < 0.001, and ****p < 0.0001; ns: no statistically significant difference.

The chemical structure of eupatilin is shown in Figure 1A. Considering its safety in the treatment of NP cells, the CCK-8 experiment evaluated eupatilin on the cytotoxicity and proliferation of NP cells. For cytotoxicity analysis, the cytotoxicity of eupatilin was calculated according to the concentration of 0-μM groups. Eupatilin showed cytotoxic effects on NP at concentrations of 25 μM or more. The number of NP cells was significantly higher at the concentration of 1.56 μM and 3.12 μM than that of 0 μM, and the difference was statistically significant (Figure 1B). For cell proliferation analysis, eupatilin at the 12.5-μM concentration range showed no significant difference in the proliferation of NP cells, the number of NP cells with a concentration of 25 μM or more was significantly lower than that of the 0 μM concentration, and the difference was statistically significant (Figures 1C–F). Based on these results, eupatilin in a concentration range of 12.5 μM was used in subsequent experiments.

### Eupatilin alleviated tumor necrosis factor-α-induced extracellular matrix degradation and improved tumor necrosis factor-α-induced senescence of nucleus pulposus cells

**FIGURE 2:**
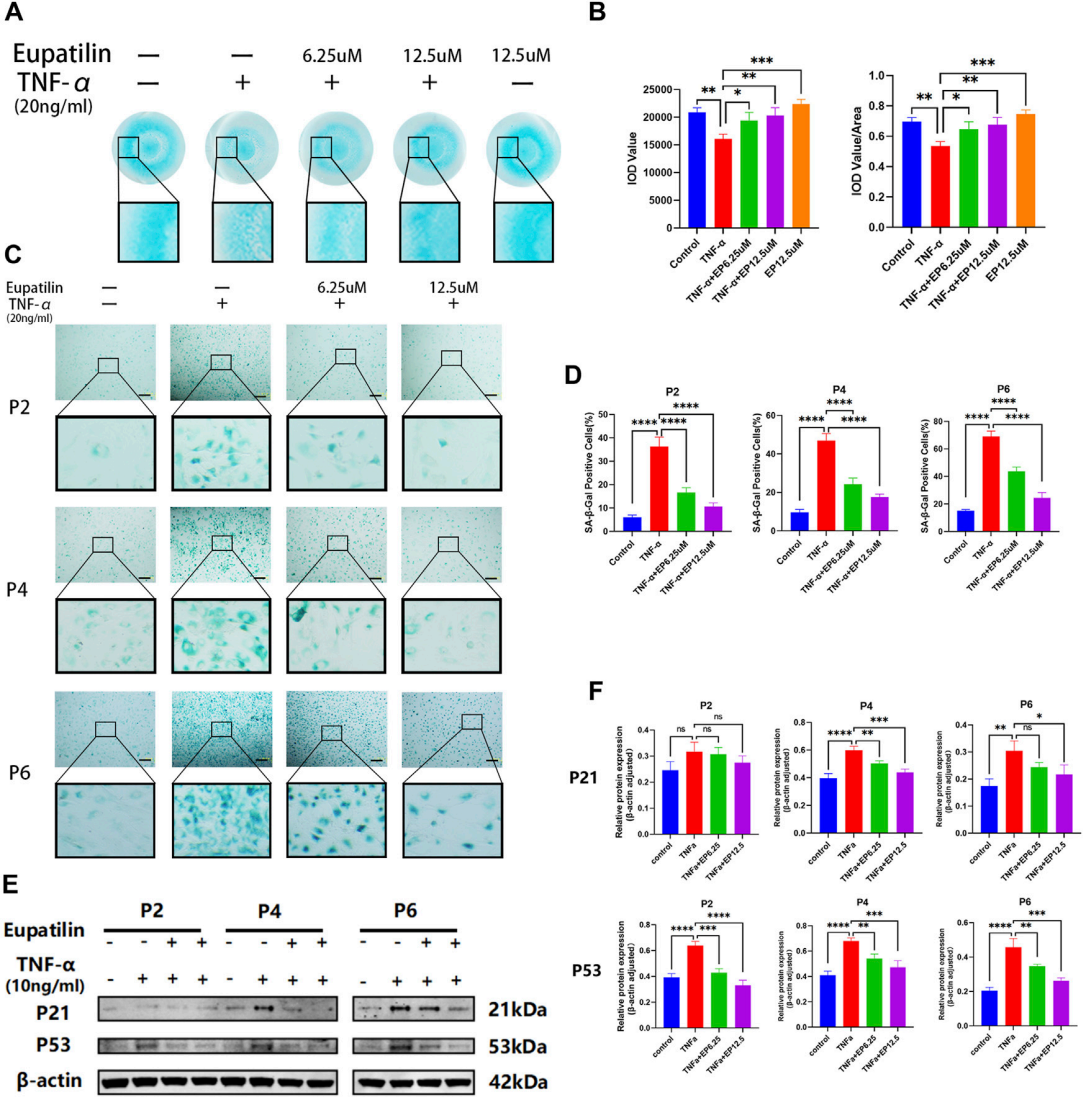
Eupatilin alleviated TNF-α-induced ECM degradation and improved TNF-α-induced senescence of NP cells. **(A)** In a high-density culture medium, after 9 days of treatment with TNF-α (20 ng/ml) or TNF-α (20 ng/ml) and eupatilin (6.25 μM, 12.5 μM), the results showed that the NP primary cells were stained with Alcian blue. Scale bar, 200 μm. **(B)** According to the results in **(A)**, the integrated optical density (IOD) value and IOD value/area calculation statistical analysis were performed to evaluate the effect of eupatilin treatment on the ECM of NP primary cells (*n* = 3). **(C)** SA-β-Gal staining results showed that the senescence changes of NP primary cells (P2, P4, and P6 generations) were treated by TNF-α (20 ng/ml) or TNF-α (20 ng/ml) and eupatilin (6.25 μM, 12.5 μM) after 3 days. Scale bar, 100 μm. **(D)** According to the results of SA-β-Gal-positive staining, NP primary cells in **(D)** were statistically analyzed (*n* = 3). **(E)** Western blot analysis showed the effects of eupatilin on p21 and p53 protein levels associated with TNF-α induced senescence of primary NP cells (P2, P4, and P6 generations). **(F)** According to the expression results of p21 and p53 in **(E)**, data are presented as the mean ± SD. *n* = 3, compared with the TNF-α-alone group. *p < 0.05, **p < 0.01, ***p < 0.001, and ****p < 0.0001; ns: no statistically significant difference.

Alcian blue staining was used to detect the effect of eupatilin on the ECM loss of TNF-α-induced primary NP cells (Figure 2A). First, the ECM loss was induced by TNF-α in high-density culture primary NP cells, and the difference was statistically significant compared with the control group. Second, eupatilin (6.25, 12.5 μM) treatment reversed the loss of ECM, and 12.5-μM concentration treatment was better than 6.25 μM; the difference was statistically significant compared with the TNF-α induction group (Figure 2B). Finally, there was no difference in Alcian blue staining in eupatilin-alone treatment group compared with the control group.

SA-β-Gal was used to detect the effect of eupatilin on the senescence of TNF-α-induced primary NP cells. As shown in Figures 2C,D, the senescence of primary NP cells (P2, P4, and P6 generations) was induced by TNF-α, and SA-β-Gal-positive NP senescent cells were significant differences between TNF-α-alone group and the control group. After the eupatilin treatment of the TNF-α-induced primary NP cells, the number of SA-β-Gal-positive NP senescent cells was significantly lower than that induced by TNF-α (Figures 2C,D). The results showed that the eupatilin treatment reversed this senescence phenomenon. Moreover, Western blot analysis was used to detect the effects of eupatilin on p21 and p53 protein levels associated with TNF-α-induced senescence of primary NP cells. The results showed that the upregulation of p21 and p53 was closely related to TNF-α induction, and those protein expression levels were significantly decreased after eupatilin treatment (Figures 2E,F). In summary, the results suggest that eupatilin attenuates TNF-α-induced ECM degradation and NP cell senescence.

### Eupatilin inhibited tumor necrosis factor-α-induced nucleus pulposus cells, upregulated metalloproteinase protein family and pro-inflammatory cytokines, downregulated SOX9 and COL2A1, and P-P65 enters the nucleus

**FIGURE 3:**
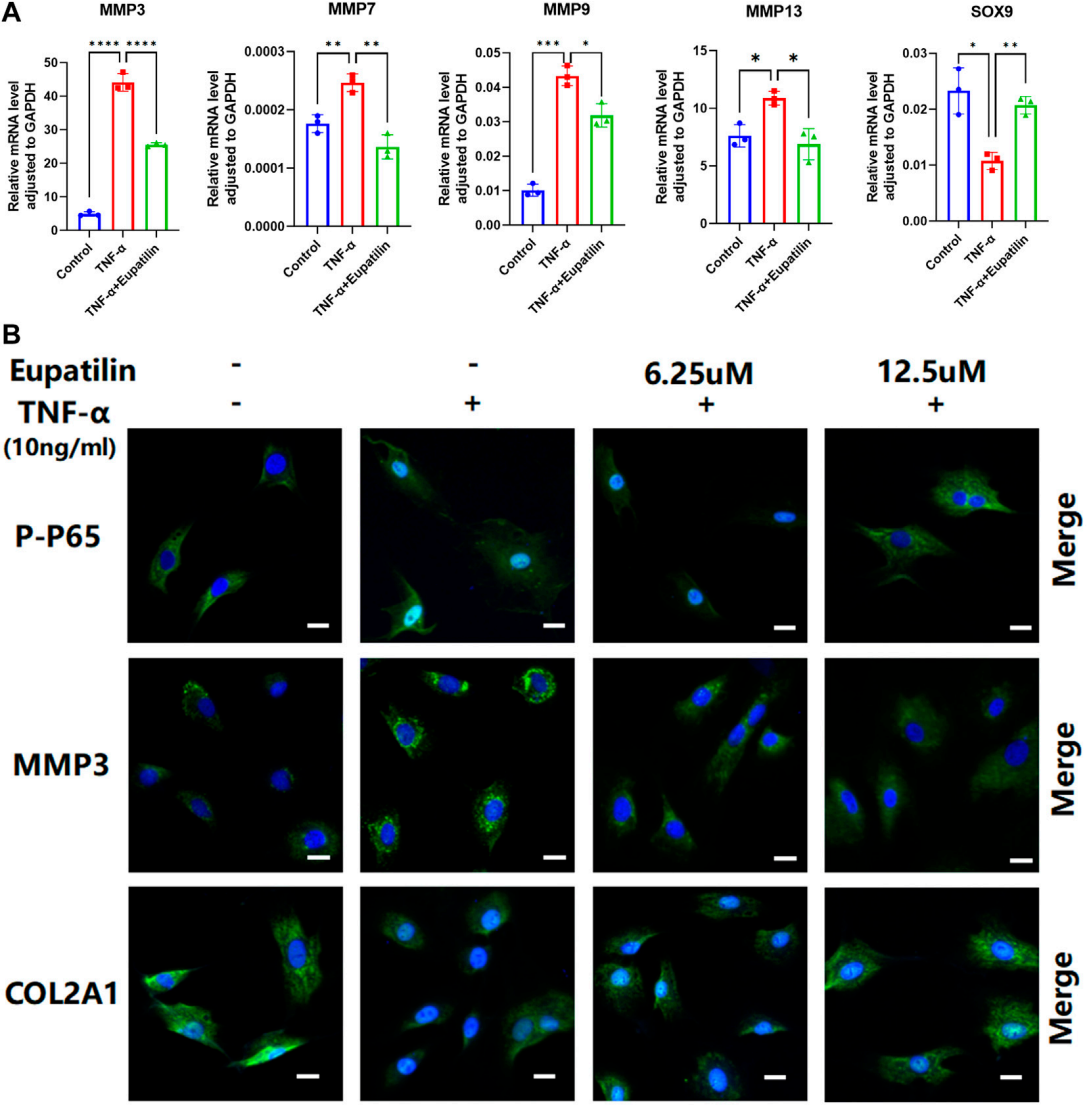
Eupatilin inhibited TNF-α-induced NP cells, upregulated the MMP family, downregulated SOX9 and COL2A1, and P-P65 enters the nucleus. **(A)** RT qPCR was used to detect the expression of anabolic and catabolic genes SOX9 containing MMP3, MMP7, MMP9, and MMP13 after treatment with TNF-α (20 ng/ml) with or without eupatilin (12.5 μM) for 24 h (*n* = 3). **(B)** Phosphorylation and nuclear translocation of NF-κB p65 were detected by immunofluorescence assay after 20 min of TNF-α (20 ng/ml) alone or 2 h of eupatilin (6.25 μM, 12.5 μM) pretreatment, followed by 20 min of TNF-α (20 ng/ml). The expression of MMP3 and COL2A1 in NP cells was detected by immunofluorescence after the treatment of TNF-α (20 ng/ml) or TNF-α (20 ng/ml) and eupatilin (6.25 μM, 12.5 μM) for 24 h. Scale bar, 20 μm. These genes were standardized for GAPDH expression. Data are presented as the mean ± SD. *n* = 3, compared with the TNF-α-alone group. *p < 0.05, **p < 0.01, ***p < 0.001, and ****p < 0.0001.

The results of high-density staining showed that the loss of ECM induced by TNF-α in primary NP cells was protected by eupatilin treatment. In order to study the mechanism of ECM degradation in primary NP cells, RT-qPCR detected changes in anabolic and catabolic genes in the extracellular matrix of primary NP cells. The results showed the upregulation of MMP7, MMP3, MMP9, MMP13, TNF-α, and IL-1β and the downregulation of SOX9 mRNA, which related to the increase in catabolism after TNF-α induction. These changes were partially reversed by eupatilin (Figure 3A and Supplementary Figures S1A,B).

In order to further study the mechanism of NP cells’ ECM degradation, we detected the expression of p-p65, MMP3, and COL2A1 in the cells by immunofluorescence. The results showed that p-p65 entering the nucleus increased significantly, the expression of MMP3 increased, and the expression of COL2A1 decreased after TNF-α induction. However, eupatilin improved these phenomena and inhibited p65 phosphorylation and translocation from the cytoplasm to the nucleus (Figure 3B). These data suggest that eupatilin attenuates TNF-α-induced degradation of ECM and senescence of NP cells by inhibiting p65 phosphorylation into the nucleus.

### Eupatilin attenuates tumor necrosis factor-α-induced extracellular matrix degradation and improves the senescence of nucleus pulposus cells *via* inhibition of the MAPK/NF-κB signaling pathway

**FIGURE 4:**
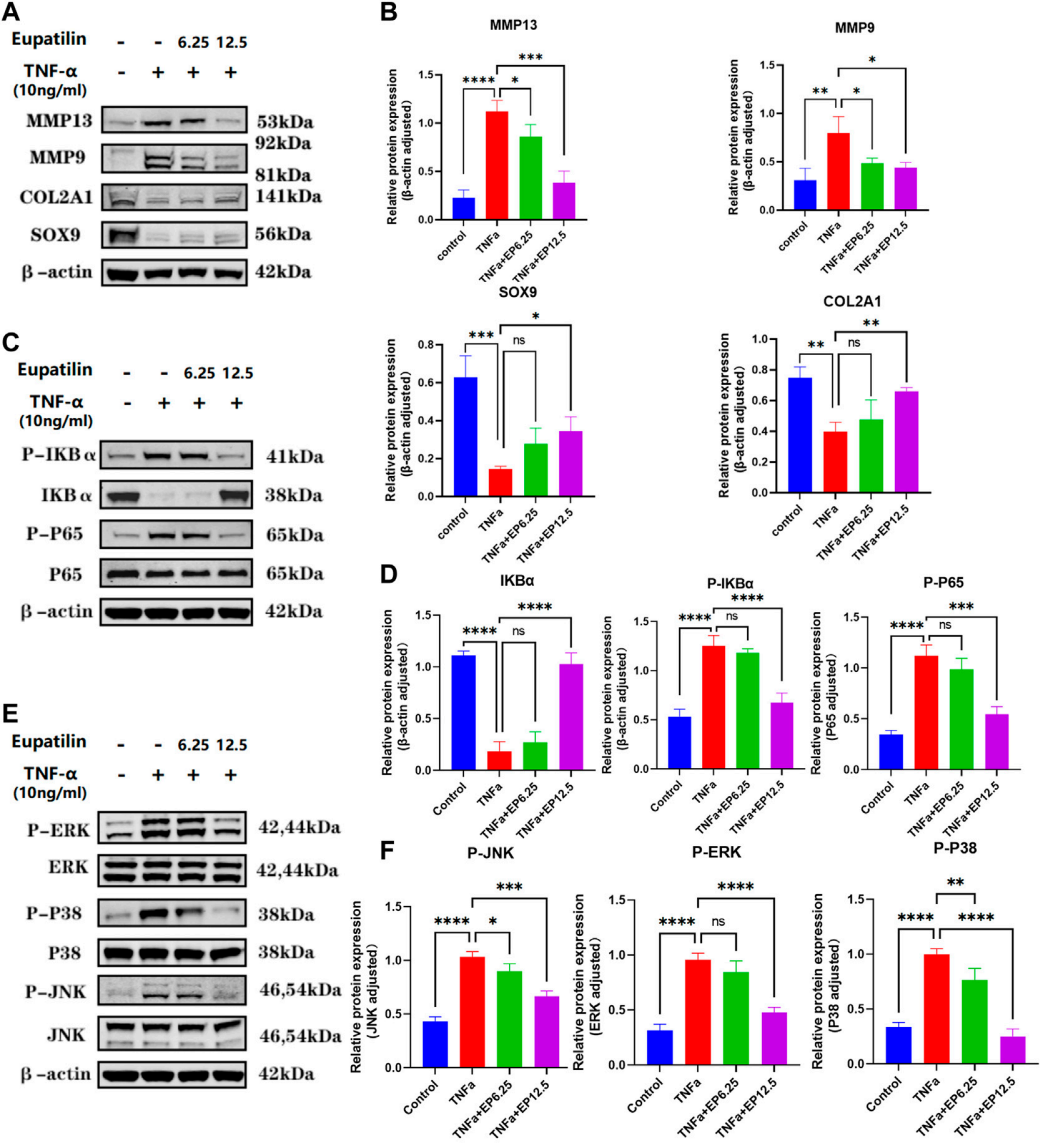
Eupatilin attenuates TNF-α-induced ECM degradation and improves the senescence of NP cells *via* inhibition of the MAPK/NF-κB signaling pathway. **(A)** Western blot was used to detect the expression of MMP9, MMP13, SOX9, and COL2A1 proteins in the cytoplasm after treatment with TNF-α (20 ng/ml) with or without eupatilin (6.25 μM, 12.5 μM) for 24 h. **(B)** According to the results, proteins in **(A)** were statistically analyzed (*n* = 3). **(C)** Western blot was performed on the effects of eupatilin on the phosphorylation of IκBα and p65, and total IκBα induced by TNF-α. NP cells were pretreated with eupatilin (6.25 μM, 12.5 μM) for 2 h before the stimulation with TNF-α (20 ng/ml) for 20 min. **(D)** Statistical analysis (*n* = 3) according to the results in **(C)**. **(E)** Western blot was performed on the effects of eupatilin on the phosphorylation of p38, RRK, and JNK induced by TNF-α. NP cells were pretreated with eupatilin (6.25 μM, 12.5 μM) for 2 h before the stimulation with TNF-α (20 ng/ml) for 20 min. **(F)** Statistical analysis (*n* = 3) according to the results in **(E)**. Data are presented as the mean ± SD. *n* = 3, compared with the TNF-α-alone group. *p < 0.05, **p < 0.01, ***p < 0.001, and ****p < 0.0001; ns: the difference was not statistically significant.

In order to study the mechanism of the effect of eupatilin on the anabolism, catabolism, and senescence of NP cells, we first performed Western blot to detect the changes of anabolic and catabolic proteins in NP cells. The results showed eupatilin can effectively alleviate the increase in MMP13 and MMP9 protein expression levels induced by TNF-α and reverse the reduction of protein in SOX9 and COL2A1 induced by TNF-α (Figures 4A,B). Second, NP cells were pretreated with eupatilin for 2 h and then stimulated with TNF-α for 20 min. Western blot showed that the phosphorylation of IκBα and p65 was upregulated by TNF-α. This indicated that NF-κB signaling was activated by TNF-α stimulation. However, eupatilin was able to inhibit these phosphorylations (Figures 4C,D). In addition, eupatilin pretreatment inhibited the degradation of IκBα induced by TNF-α (Figure 4C).

TNF-α also activates another inflammatory pathway, the MAPK pathway. Western blot showed that p38, JNK, and ERK phosphorylation were upregulated after 20 min of TNF-α stimulation. Interestingly, eupatilin also inhibited the phosphorylation of MAPK signaling (Figures 4E,F). These findings suggest that eupatilin can improve the degradation of NP cell ECM by inhibiting TNF-α-induced activation of NF-κB and MAPK pathways, thus reducing the senescence of NP cells.

### Administration of eupatilin ameliorated the progression of the puncture-induced model of intervertebral disc degeneration *in vivo*

**FIGURE 5:**
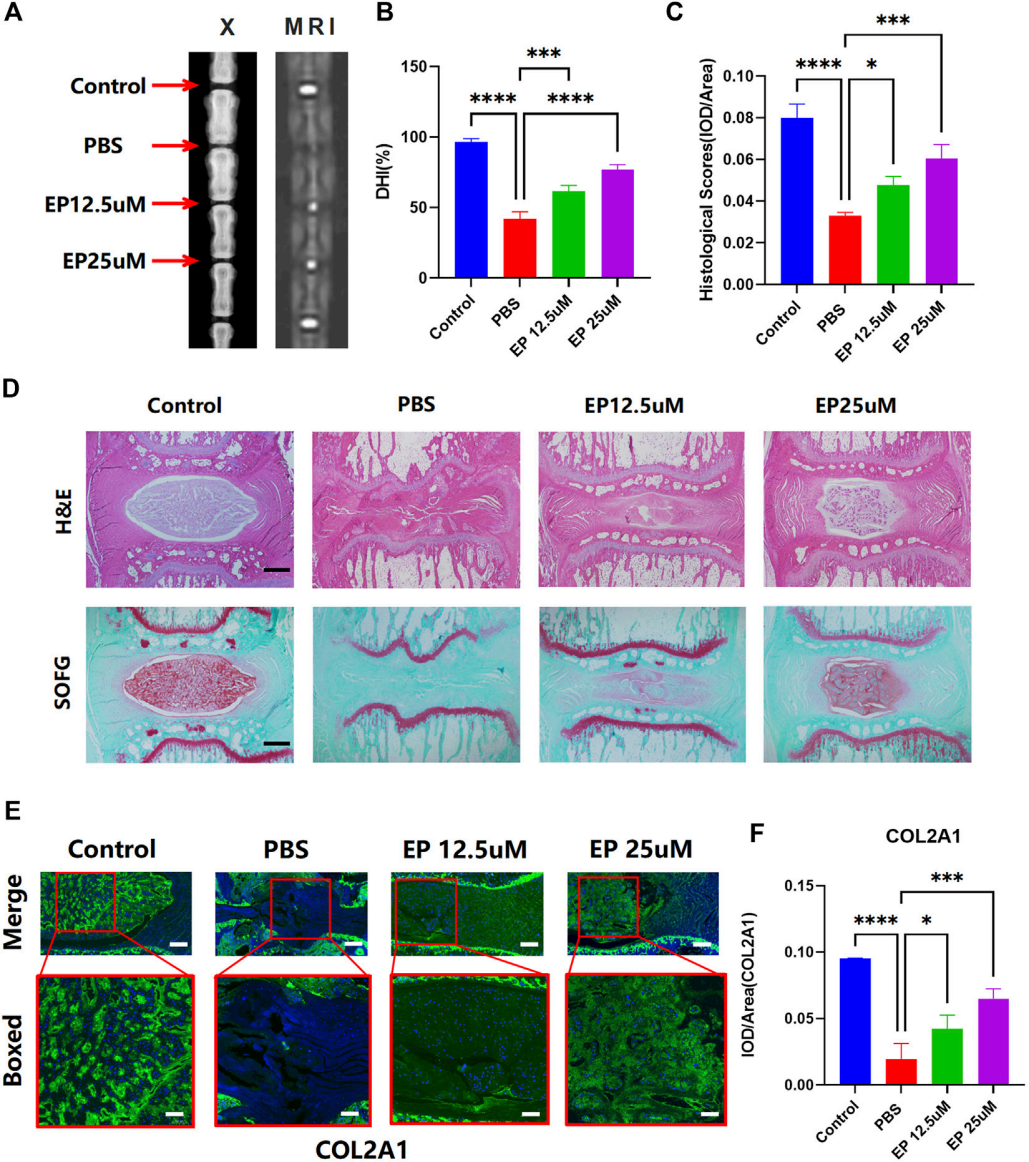
Administration of eupatilin ameliorated the progression of the puncture-induced model of IDD *in vivo*. **(A)** Puncture-induced model of IDD treated for 4 weeks with or without eupatilin were scanned with animal X-ray and MRI in the rat. **(B)** Quantitative statistical analysis (*n* = 3) according to the result of **(A)** of the intervertebral disc height index (DHI, %). **(D)** IVD of hematoxylin and eosin staining and safranin O-Fast green staining results. Scale bar, 500 μm. **(C)** Histological score was statistically analyzed according to the results in **(D)** (*n* = 3). **(E)** Results of COL2A1 immunofluorescence staining of IVD. Scale bar, 500 μm. **(F)** IOD value/area was statistically analyzed, according to the results in **(E)**. Data are presented as the mean ± SD. *n* = 3, compared with the PBS group. *p < 0.05, ***p < 0.001, and ****p < 0.0001.

Given the reduced ECM degradation and improved cell senescence effect *in vitro*, we further examined whether eupatilin can improve the progress of IDD *in vivo*. We first examined the animal X-ray, MRI, and DHI data (measured in percentages) in rats treated with or without eupatilin for 4 weeks after the puncture-induced model of caudal IDD. In the IDD model, the loss of the intervertebral height increased significantly. However, image results showed that the loss of the intervertebral height was partially improved when eupatilin was injected intravenously into the caudal IVD (Figures 5A,B). Safranin O-Fast green staining, and hematoxylin and eosin staining of IVDs from each group showed that eupatilin significantly reduced the destruction of the disc structure compared with the PBS group (Figures 5C,D). Immunofluorescence staining for COL2A1 was performed to examine the effect of eupatilin on ECM *in vivo* (Figure 5E). The results showed that eupatilin could ameliorate the degradation of COL2A1 in the rat caudal intervertebral disc, which was consistent with the results *in vitro* (Figure 5F). These results suggest that eupatilin has potential therapeutic effects on the development of IDD in rats.

## Discussion

IDD is the main cause of low back pain. There is increasing evidence that NP cell senescence and extracellular matrix degradation are the key factors of IDD and are positively correlated with the progress of IDD (Li et al., 2021; Novais et al., 2021). Furthermore, some studies have shown that inflammation may lead to NP cell senescence and ECM loss (Li et al., 2017a; Xie et al., 2018). In this study, we demonstrated that TNF-α significantly promotes NP cell senescence and ECM degradation, which is consistent with previous studies.

There is a gelatinous NP tissue at the center of the IVD that not only maintains disc height but also meets the mechanical requirements of the body to bend and release axial mechanical stress (Feng et al., 2018). IDD is associated with anabolism and catabolism of the NP ECM. The increase of cell senescence and inflammatory cytokines during the progression of IDD breaks the metabolic balance of the ECM, and the metabolic dysfunction of NP cells eventually leads to the loss of type II collagen and proteoglycan in NP tissue (Wang et al., 2018b). The height of IVD decreased with the decrease in the number of functional NP cells in IVD and the accumulation of senescent cells. Senescent NP cells have strong metabolic activity, with increased secretion of inflammatory cytokines and MMPs that accelerate the degradation of the ECM (Novais et al., 2019; Li et al., 2021). This phenomenon is known as the senescence-associated secretory phenotype. The senescence of NP cells and the increase in inflammatory cytokines lead to the vicious circle of NP cell degeneration and accelerate the development of IDD (Ashraf et al., 2021). Therefore, drugs with anti-inflammatory and anti-aging therapeutic effects may prevent the occurrence of IDD and delay the progress of IDD.

Eupatilin is a kind of flavonoid with pharmacological activity extracted from *Artemisia argyi*, which has anti-inflammatory and anti-oxidation properties (Nageen et al., 2020). Eupatilin has been reported to significantly inhibit allergic inflammation through NF-κB and MAPK signaling pathways (Song et al., 2018), inhibit TNF-α-induced MMP expression (Jung et al., 2017), and inhibit oxidative damage and promote the formation of the ECM in chondrocytes (Jeong et al., 2015). Our results suggest that eupatilin can improve TNF-α-induced ECM degradation and cell senescence through MAPK and NF-κB signaling pathways. Meanwhile, *in vivo* experiments confirmed that eupatilin treatment can effectively improve the progress of IDD. TNF-α is an important inflammatory factor that activates MAPK and NF-κB signaling pathways (Lv et al., 2021). Furthermore, TNF-α is closely related to the degeneration of IVD tissue, mediates ECM degradation, and leads to cell senescence by activating MMPs and degrading proteoglycan and collagen type II (Ashraf et al., 2021). Therefore, blocking the activation of the signal pathway induced by TNF-α can effectively prevent the occurrence and development of IDD.

First, TNF-α could significantly promote the ECM degradation of NP cells, but eupatilin could reverse the ECM degradation induced by TNF-α. Meanwhile, eupatilin partially reversed TNF-α-induced upregulation of MMP9 and MMP13 and downregulation of SOX9 and COL2A1 at protein and transcriptional levels. These data show that eupatilin has a protective effect on the degradation of the ECM, which has been consistent with previous research results. Second, cell senescence is another key factor in the development of IDD. SA-β-Gal staining, p21, and p53 are reliable and classic markers of cell senescence (Chen et al., 2020; Qiu et al., 2020). Our data showed that eupatilin significantly reduced the number of SA-β-gal-positive cells and downregulated the expression of p53 and p21 proteins. This indicates that eupatilin can effectively attenuate TNF-α-induced NP cell senescence. Finally, NF-κB and MAPK signaling pathways have been reported to play an important role in TNF-α-induced NP cell senescence and ECM degradation (Chen et al., 2021; Kang et al., 2022). The relationships between these signaling pathways form a vicious cycle that accelerates the progress of IDD. TNF-α triggers the activation of the NF-κB pathway, including the phosphorylation of IKKα, IκBα, and p65, as well as the degradation of IκBα, which initiates upregulation of downstream catabolism enzymes and inflammatory cytokines, leading to ECM degradation and cell senescence. Our data showed that eupatilin significantly inhibited the phosphorylation of IκBα and p65 in TNF-α-induced NP cells and prevented the degradation of IκBα. In addition, the activation of the MAPK pathway, including the phosphorylation of p38, JNK, and ERK, is also involved in the regulation of inflammatory response, which is closely related to cell senescence and ECM degradation in the progress of IDD (Chen et al., 2021). Surprisingly, our data showed that eupatilin significantly downregulated TNF-α-induced phosphorylation of JNK, p38, and ERK.

**FIGURE 6:**
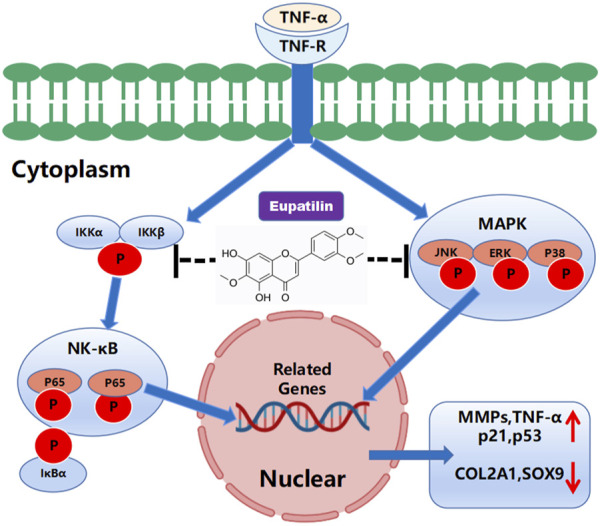
Schematic representation of the molecular mechanism of eupatilin in TNF-α-induced activation of inflammatory signaling pathways and NP cell senescence, in the anabolism and catabolism of NP cell extracellular matrix.

In this study, we investigated the anti-inflammatory and anti-senescence effects of eupatilin on TNF-α-induced NP cells. We observed that the expression of MMPs, P21, and P53 increased after TNF-α stimulated NP cells, while the expression of COL2A1 and SOX9 decreased. High-density staining showed ECM degradation and the increase of SA-β-gal-positive cells of P2–P6 generation. When eupatilin was combined with TNF-α, this phenomenon was suppressed. In mechanism, TNF-α activates NF-κB and MAPK signaling pathways, leading to NP cell senescence, inflammatory factor secretion, and ECM degradation. After eupatilin treatment, it inhibited the increase of the expression of MMPs, p21, and p53 in NP cells and the decrease of the expression of COL2A1 and SOX9, so as to adjust ECM the dynamic balance between anabolism and catabolism, finally reduce the degradation of the ECM, improve the senescence of NP cells, and effectively slow down the progress of IDD. These results confirm our previous hypothesis that MAPK and NF-κB signal transduction generate a vicious cycle of inflammatory cascades that accelerate IDD (Figure 6).

In conclusion, our results confirmed that eupatilin can inhibit MAPK and NF-κB signal transduction, reduce TNF-α-induced degradation of ECM, and improve the senescence of NP cells. Also, our *in vivo* data showed that eupatilin plays a protective role in the progression of IDD. Therefore, eupatilin can be considered as a potential therapeutic strategy for IDD based on the results of this study *in vivo* and *in vitro*.

## Data Availability

The raw data supporting the conclusion of this article will be made available by the authors, without undue reservation.
