# Supplementary material for: Eupatilin attenuates the senescence of nucleus pulposus cells and mitigates intervertebral disc degeneration via inhibition of the MAPK/NF-κB signaling pathway
Source: Front Pharmacol. 2022 Nov 3;13:940475. doi: 10.3389/fphar.2022.940475 (PMC9669913; doi:10.3389/fphar.2022.940475)
Supplement: Supplementary file 1 [file Table1.DOCX]

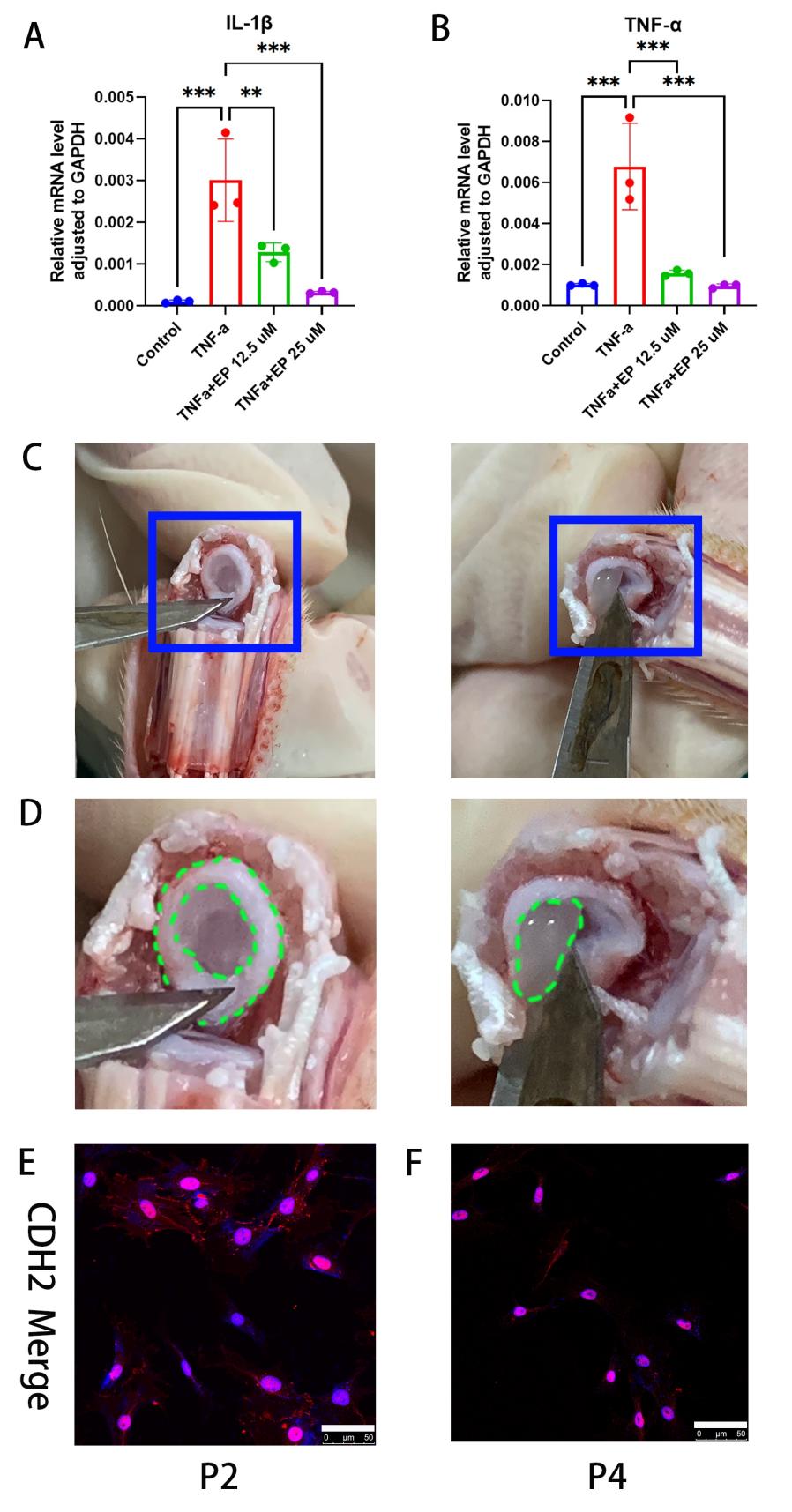


**Supplementary Fig. EP inhibited TNF-α-induced NP cells pro-inflammatory cytokines up-regulated. NP primary cells were extracted and the CDH2 marker genes of NP primary cells were identified.** (A-B) RT-qPCR was used to detect the expression level of gene TNF-α and IL-1β in NP cells after treatment with TNF-α(20 ng/ml) with or without Eupatilin for 24 h (n=3). (C) Schematic diagram of NP primary cells extracted from rat intervertebral disc. (D) An enlarged view of the blue box in Figure C. (E-F) Immunofluorescence was used to identify the expression of marker gene CDH2 in NP cells during their passage. Data are presented as mean ± SD, **P＜0.01, ***P＜0.001
