# Supplementary figures and images for: Eupatilin attenuates the senescence of nucleus pulposus cells and mitigates intervertebral disc degeneration via inhibition of the MAPK/NF-κB signaling pathway
Source: Front Pharmacol. 2022 Nov 3;13:940475. doi: 10.3389/fphar.2022.940475 (PMC9669913; doi:10.3389/fphar.2022.940475)

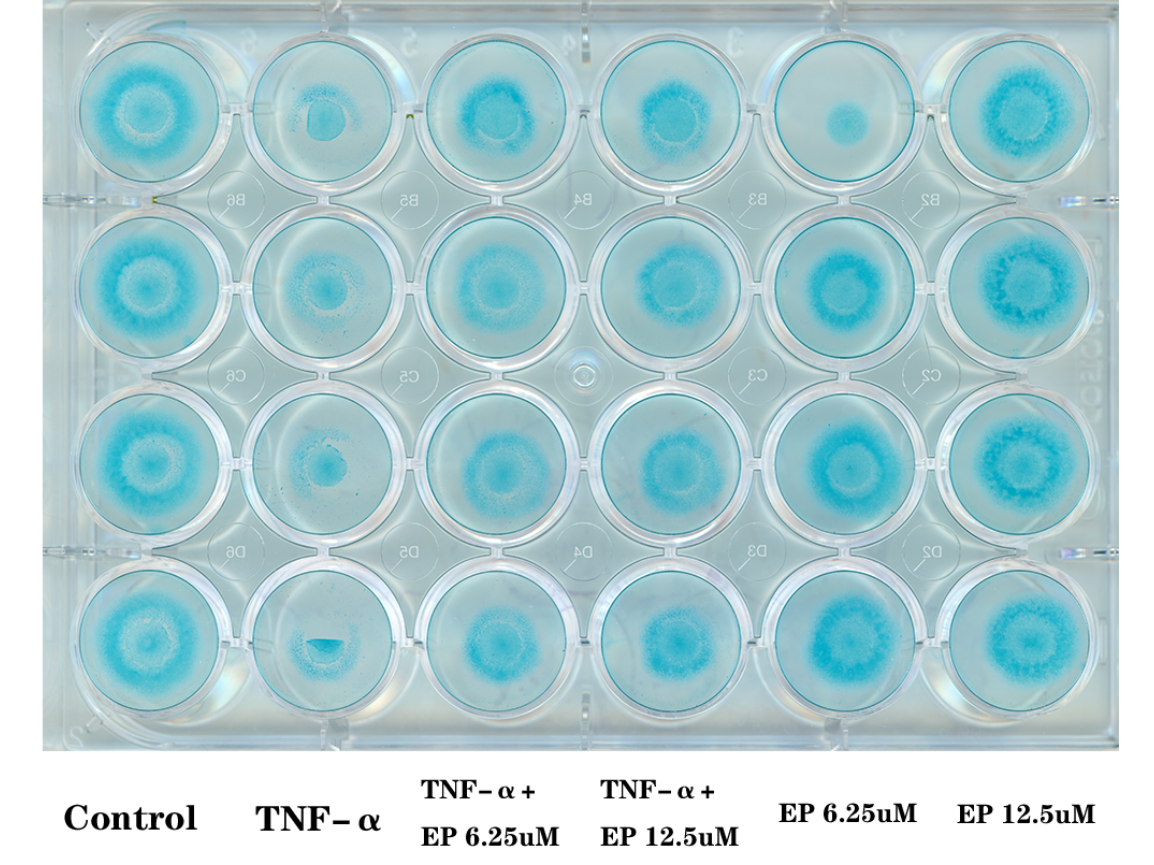

Supplement: Supplementary file 2 [file DataSheet1.ZIP › Raw Data/Figure 2 original data/A/High-Density Culture and Alcian Blue Stain.bmp]

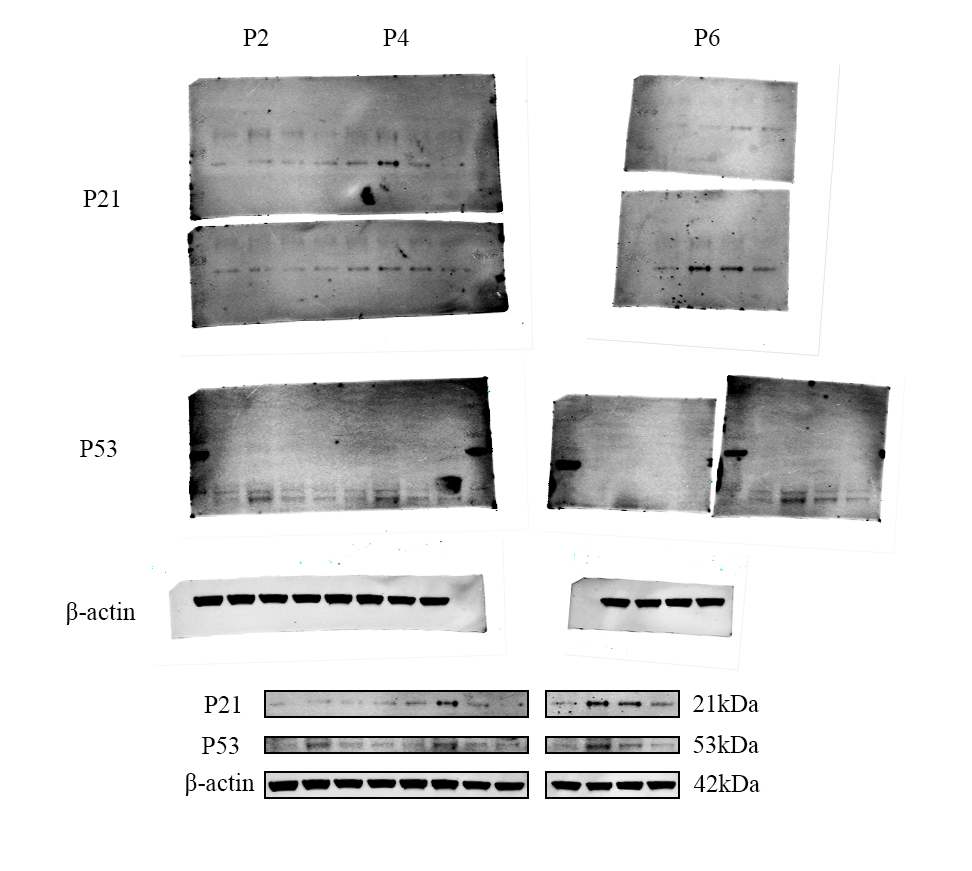

Supplement: Supplementary file 2 [file DataSheet1.ZIP › Raw Data/Figure 2 original data/E and F/WB P21 P53 .jpg]

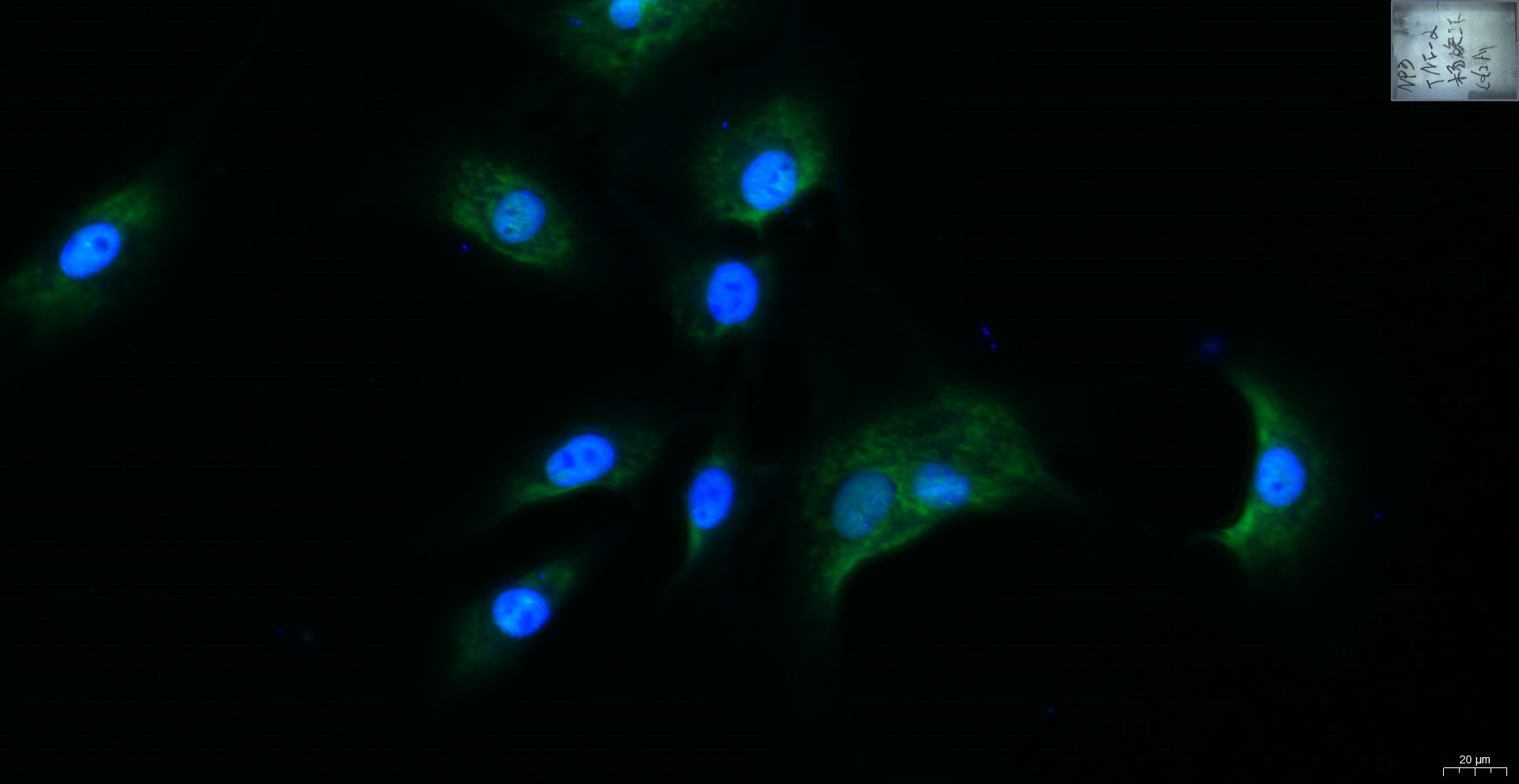

Supplement: Supplementary file 2 [file DataSheet1.ZIP › Raw Data/Figure 3 original data/B/COL2A1/TNF-a┴ IF COL-II_40.0x.jpg]

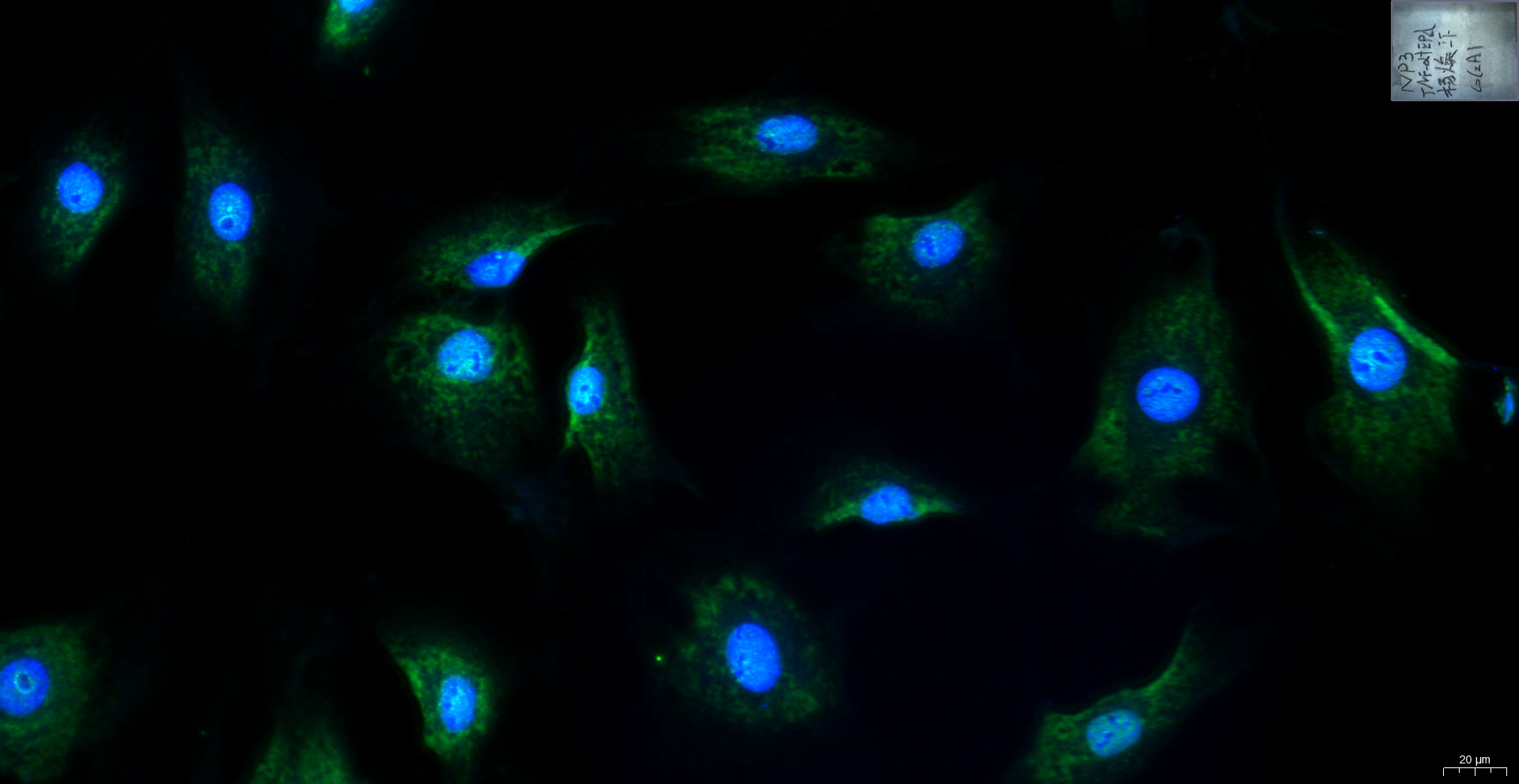

Supplement: Supplementary file 2 [file DataSheet1.ZIP › Raw Data/Figure 3 original data/B/COL2A1/TNF-a┴+EPd IF COL-II_40.0x.jpg]

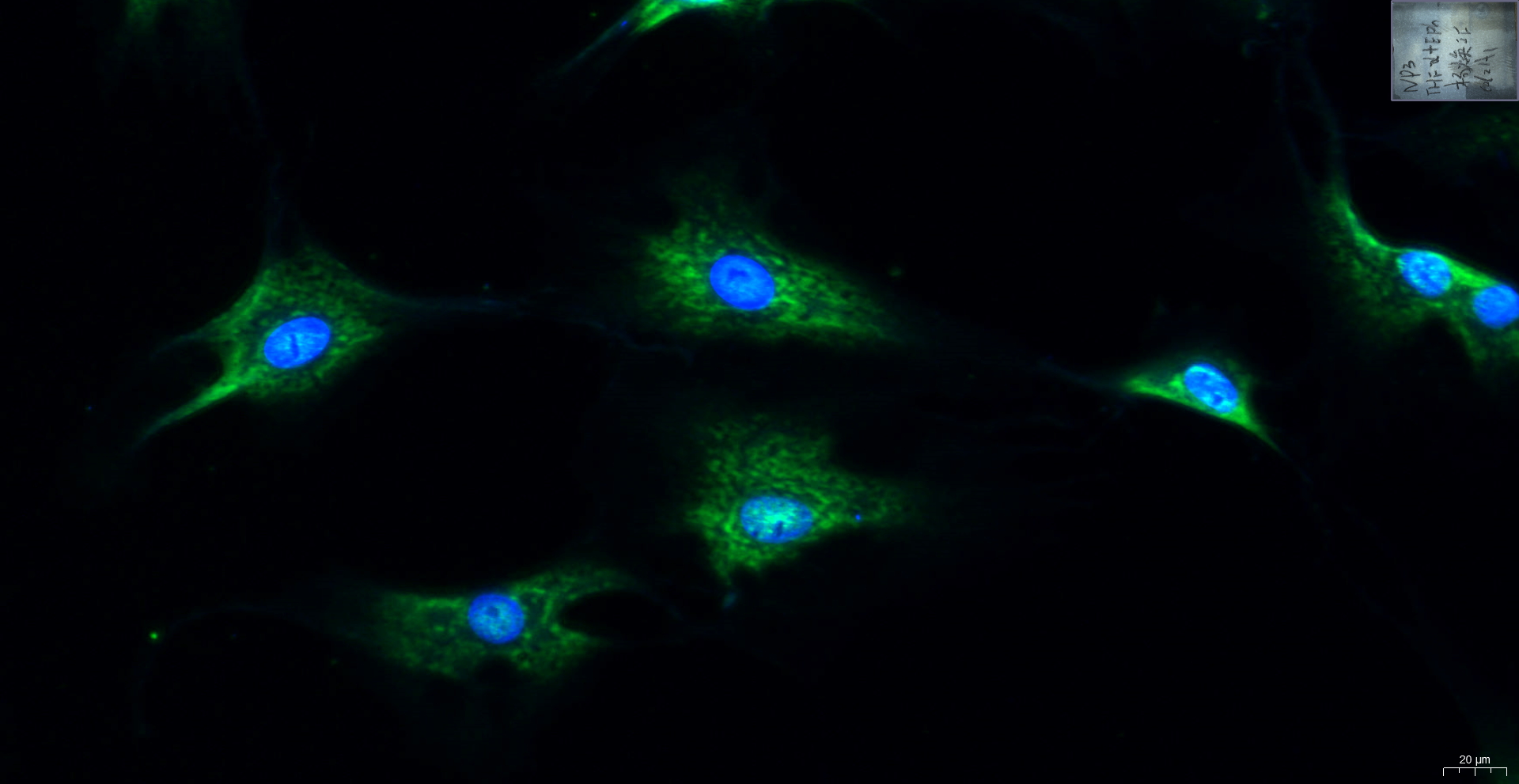

Supplement: Supplementary file 2 [file DataSheet1.ZIP › Raw Data/Figure 3 original data/B/COL2A1/TNF-a┴+EPh IF COL-II_40.0x.jpg]

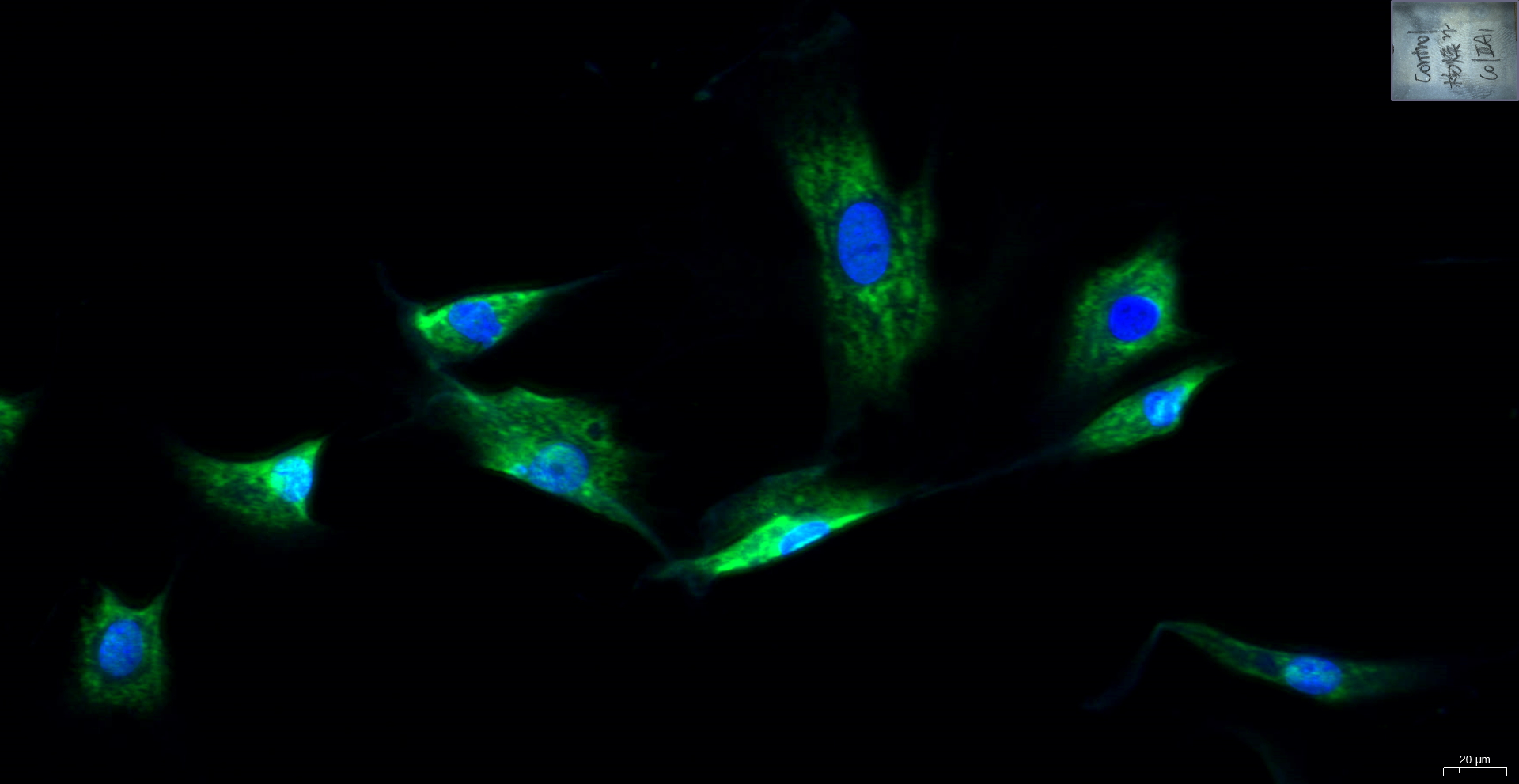

Supplement: Supplementary file 2 [file DataSheet1.ZIP › Raw Data/Figure 3 original data/B/COL2A1/control IF COL-II_40.0x.jpg]

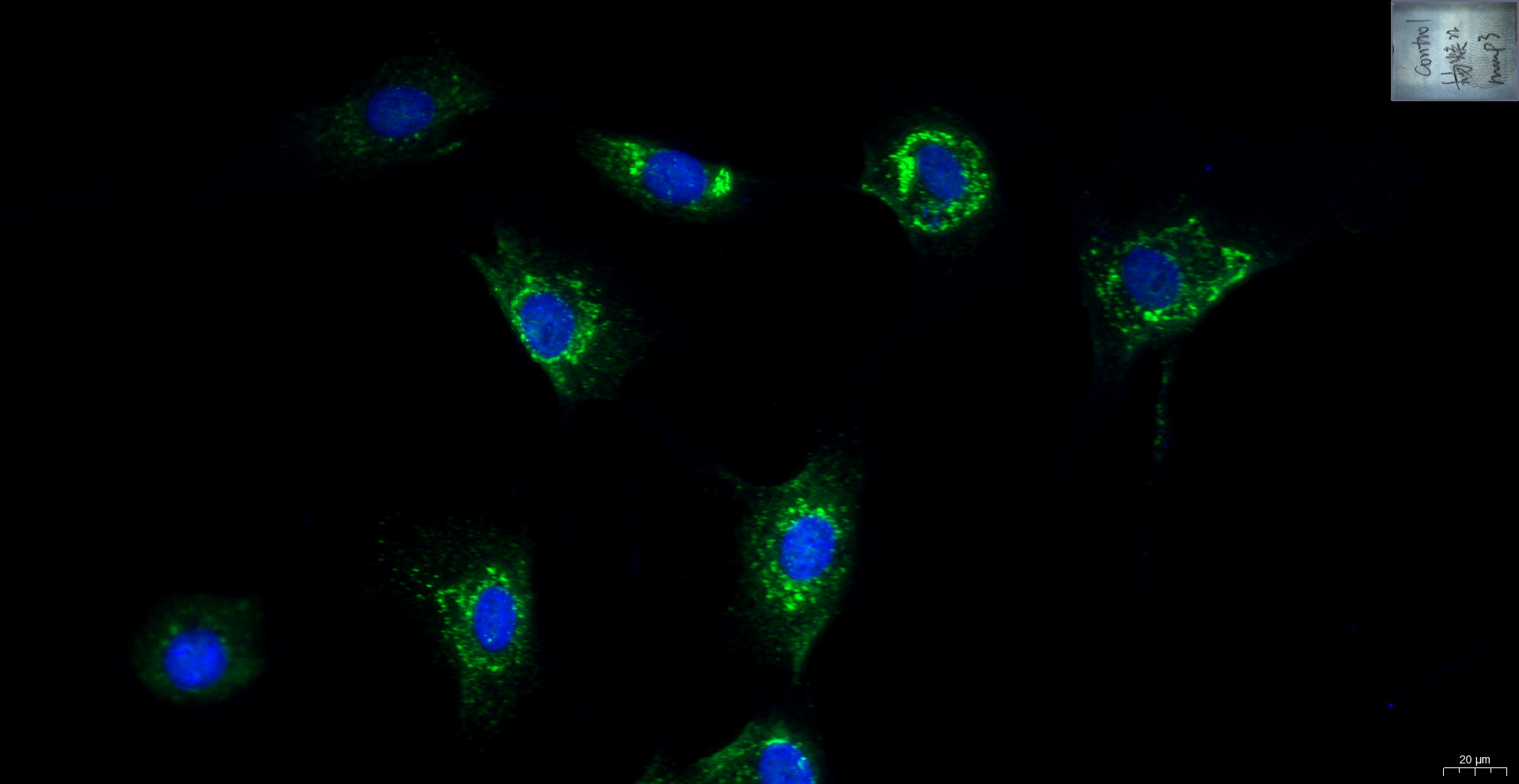

Supplement: Supplementary file 2 [file DataSheet1.ZIP › Raw Data/Figure 3 original data/B/MMP3/TNF-a IF mmp3_40.0x.jpg]

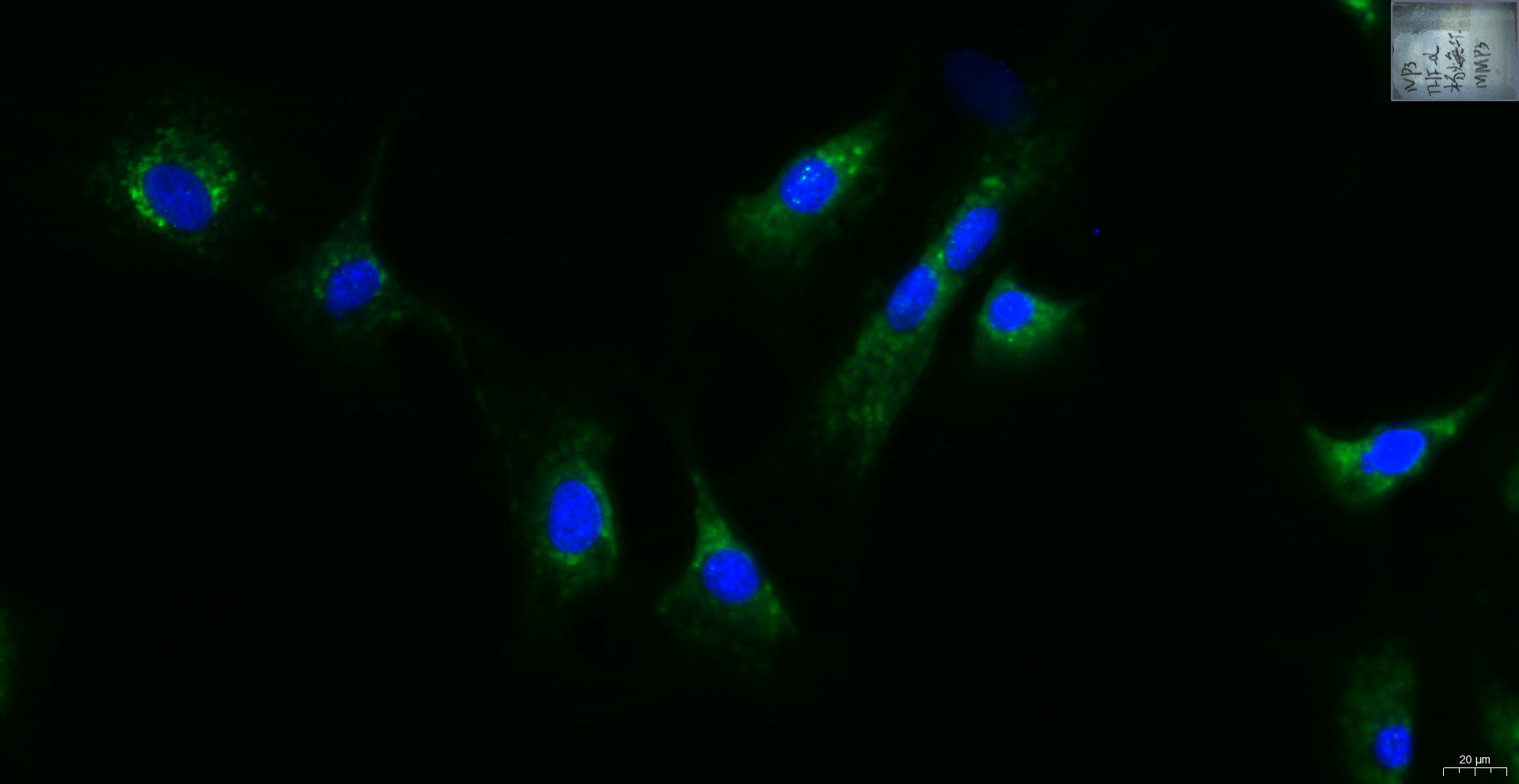

Supplement: Supplementary file 2 [file DataSheet1.ZIP › Raw Data/Figure 3 original data/B/MMP3/TNF-a┴ EP 6.25uM mmp3_40.0x.jpg]

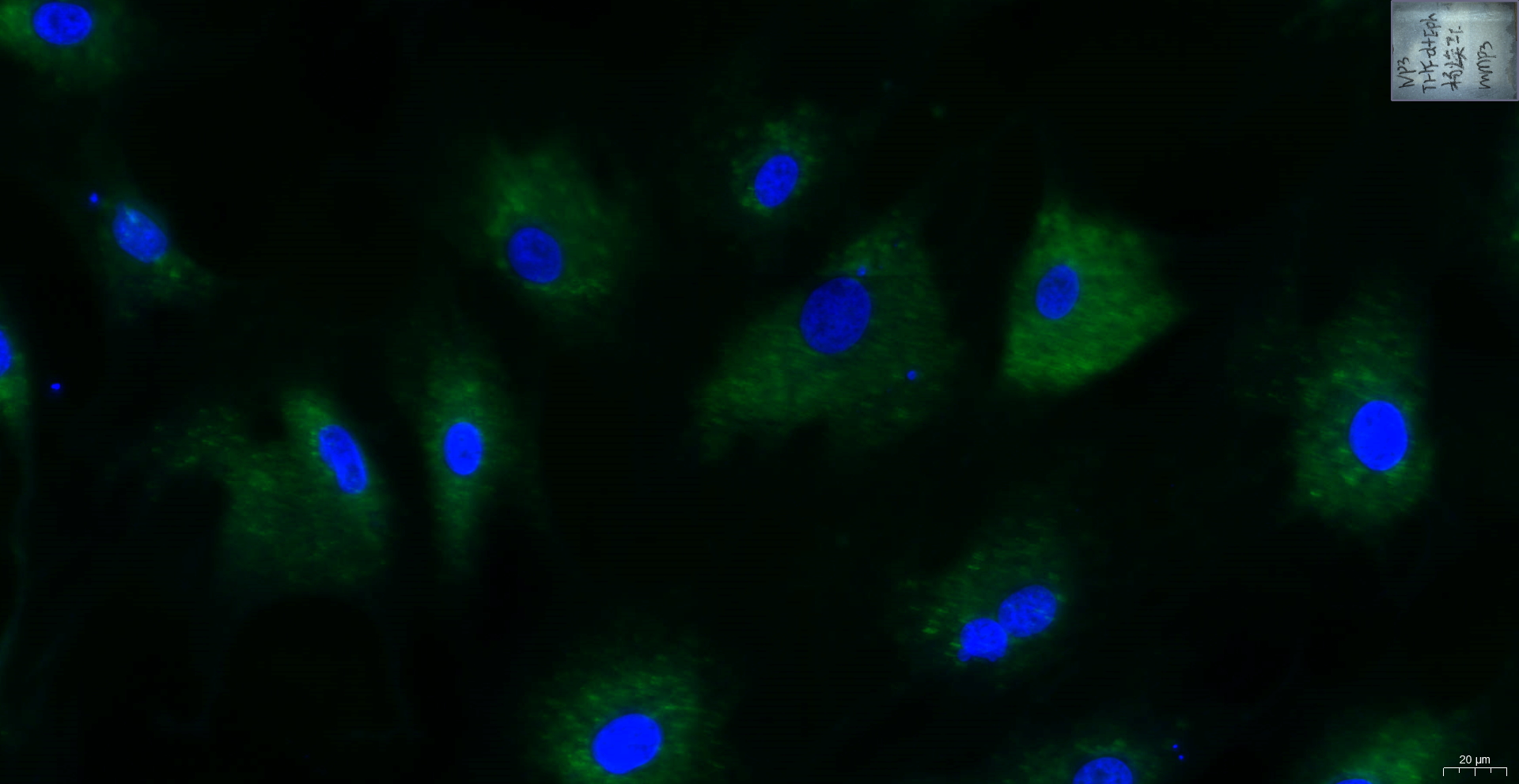

Supplement: Supplementary file 2 [file DataSheet1.ZIP › Raw Data/Figure 3 original data/B/MMP3/TNF-a┴+EP12.5 uM mmp3_40.0x.jpg]

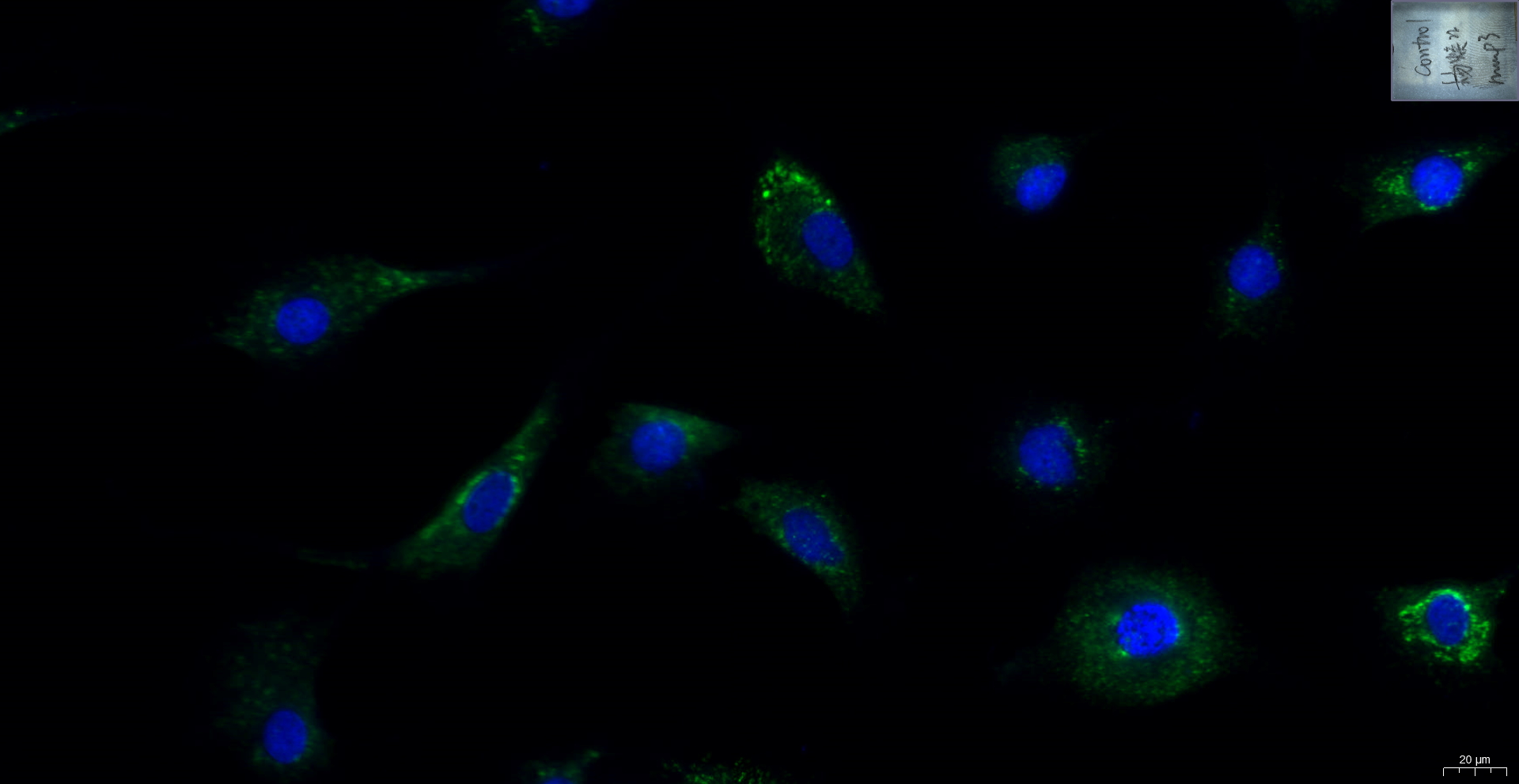

Supplement: Supplementary file 2 [file DataSheet1.ZIP › Raw Data/Figure 3 original data/B/MMP3/control IF mmp3_40.0x.jpg]

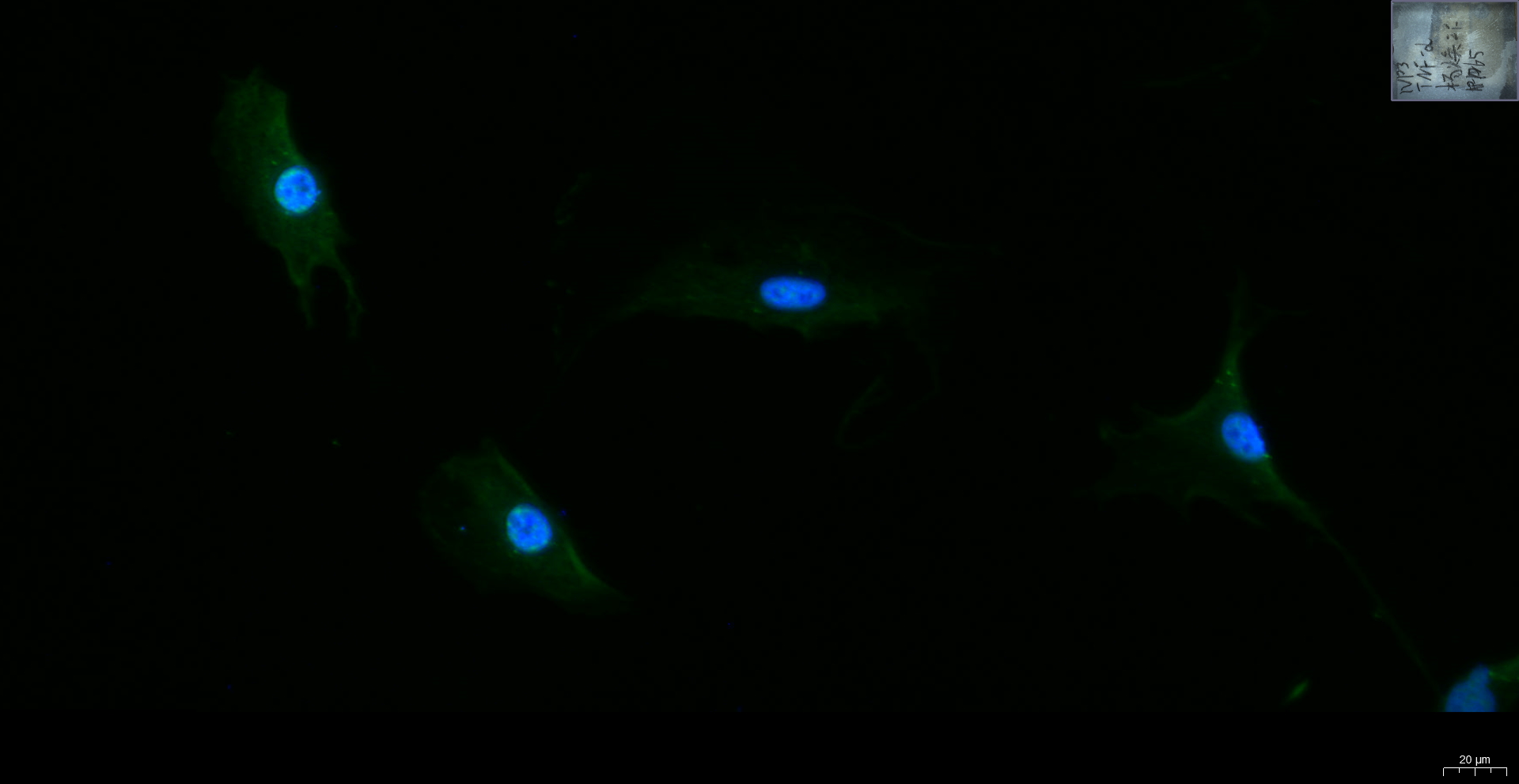

Supplement: Supplementary file 2 [file DataSheet1.ZIP › Raw Data/Figure 3 original data/B/P-P65/TNF-a┴ EP 6.25uM IF P-P65_40.0x.jpg]

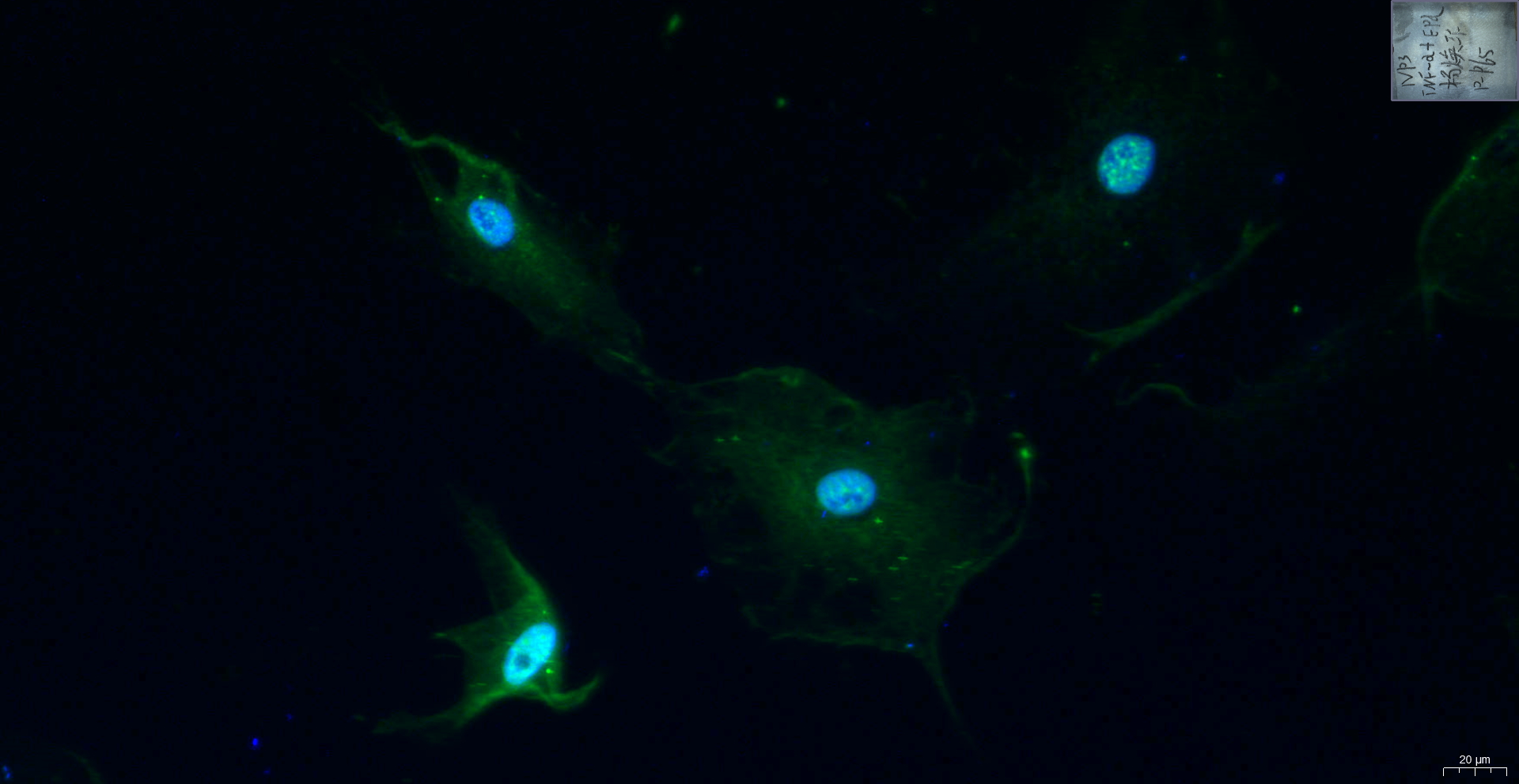

Supplement: Supplementary file 2 [file DataSheet1.ZIP › Raw Data/Figure 3 original data/B/P-P65/TNF-a┴ IF P-P65_40.0x.jpg]

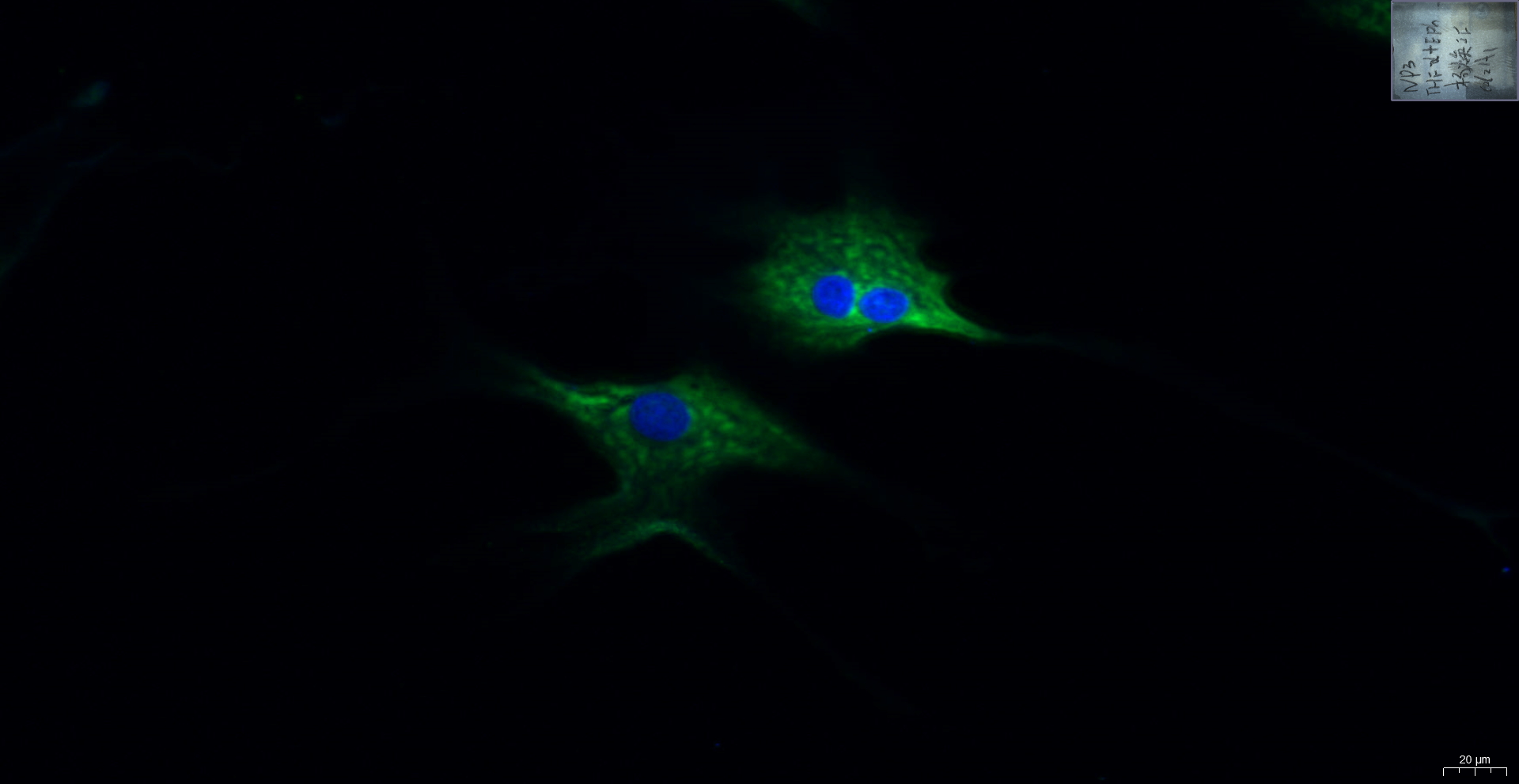

Supplement: Supplementary file 2 [file DataSheet1.ZIP › Raw Data/Figure 3 original data/B/P-P65/TNF-a┴+EP12.5 uM IF PP65_40.0x.jpg]

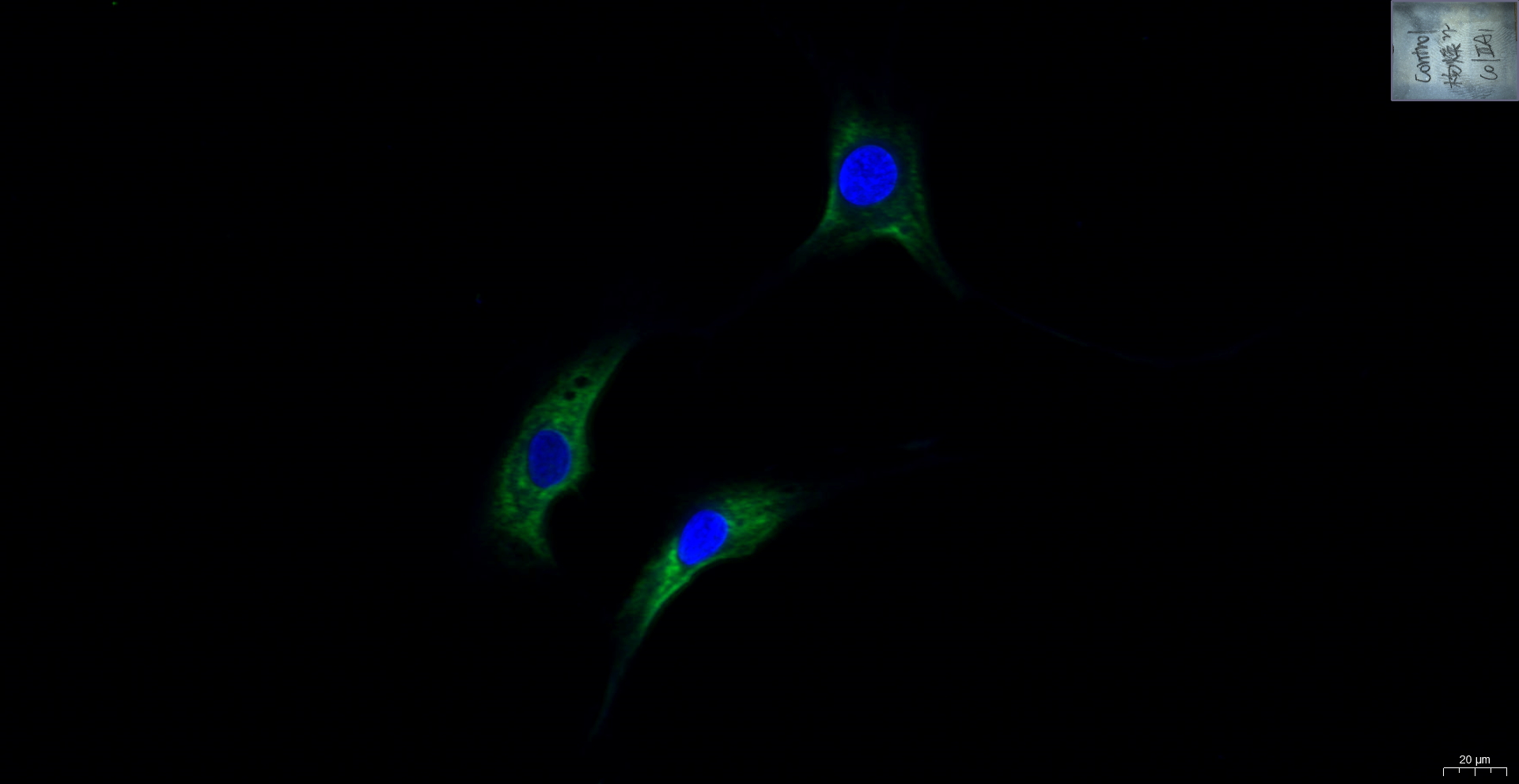

Supplement: Supplementary file 2 [file DataSheet1.ZIP › Raw Data/Figure 3 original data/B/P-P65/control IF PP65_40.0x.jpg]

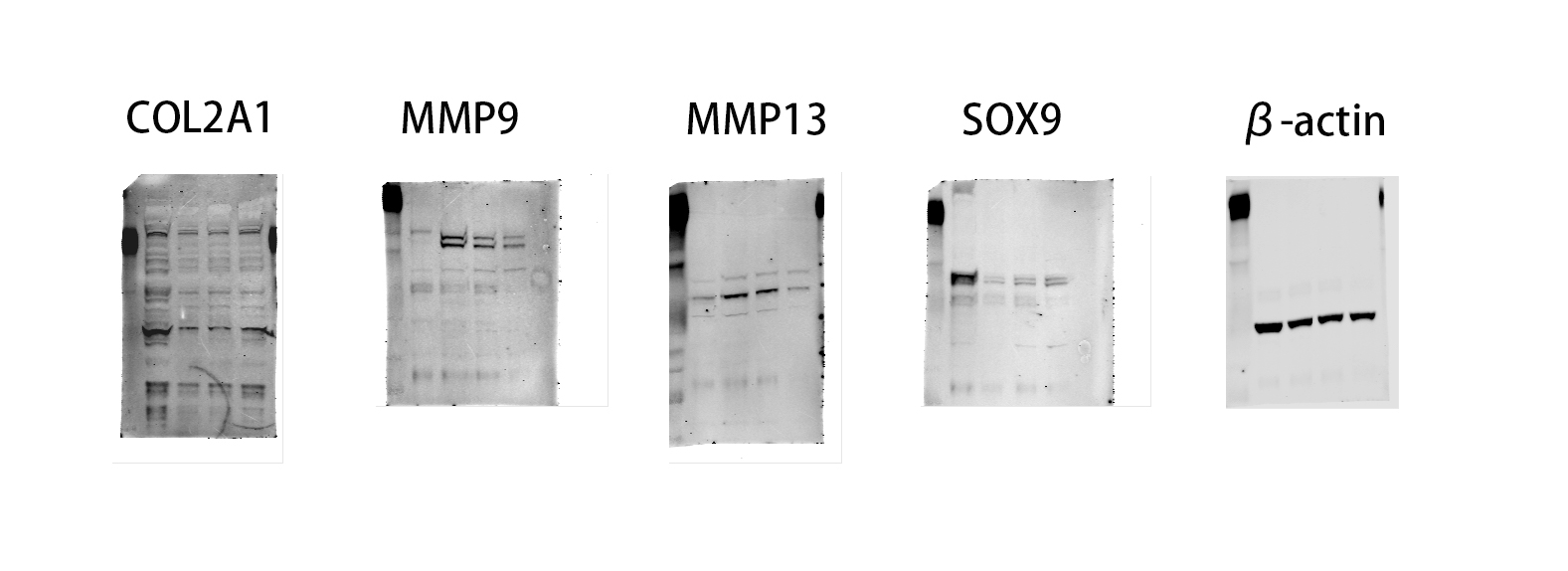

Supplement: Supplementary file 2 [file DataSheet1.ZIP › Raw Data/Figure 4 original data/A.jpg]

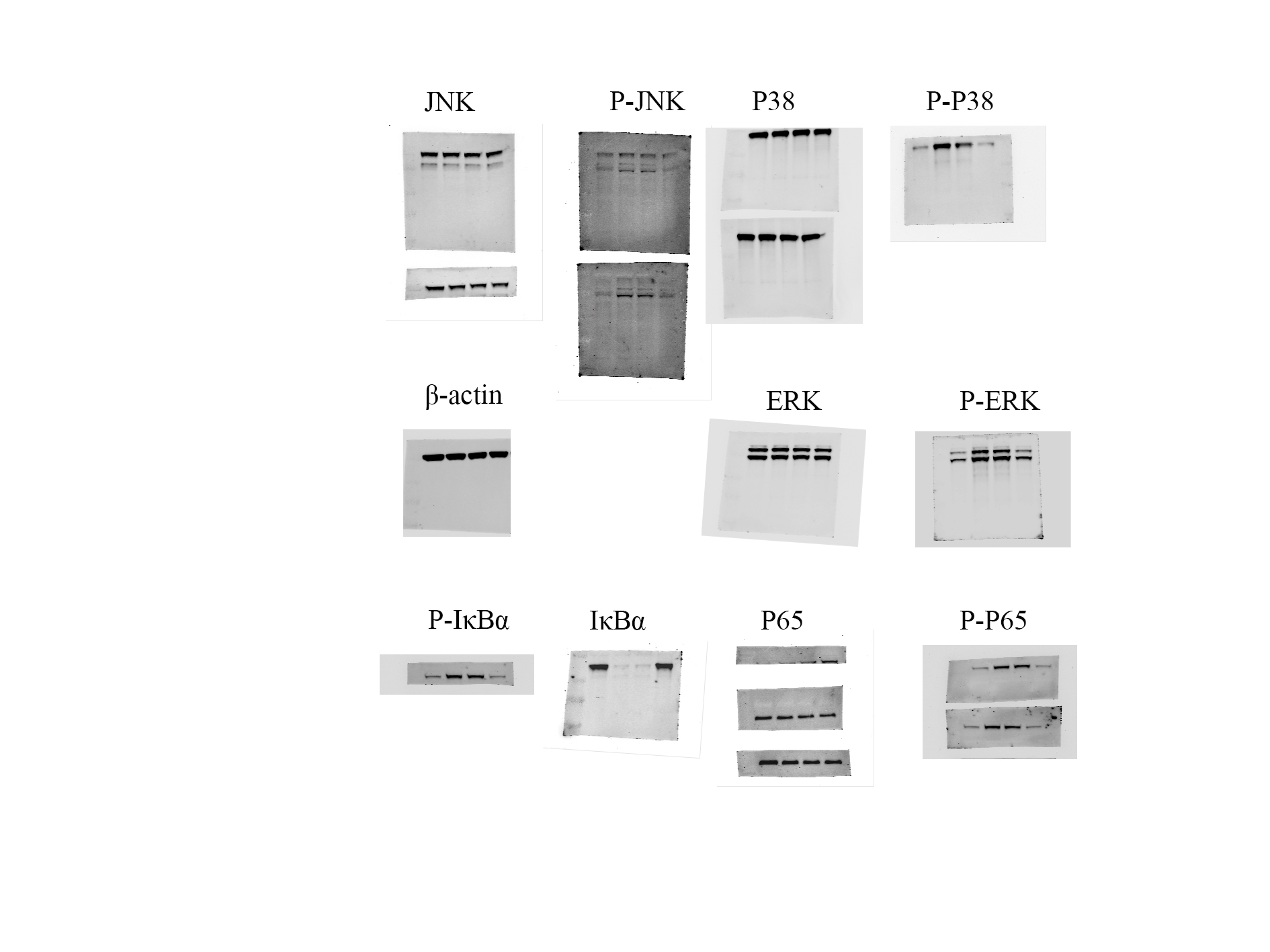

Supplement: Supplementary file 2 [file DataSheet1.ZIP › Raw Data/Figure 4 original data/C and E.jpg]

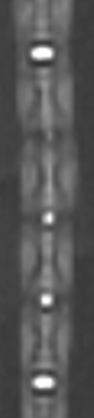

Supplement: Supplementary file 2 [file DataSheet1.ZIP › Raw Data/Figure 5 original data/A/MRI.bmp]

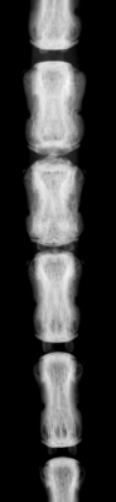

Supplement: Supplementary file 2 [file DataSheet1.ZIP › Raw Data/Figure 5 original data/A/X.bmp]

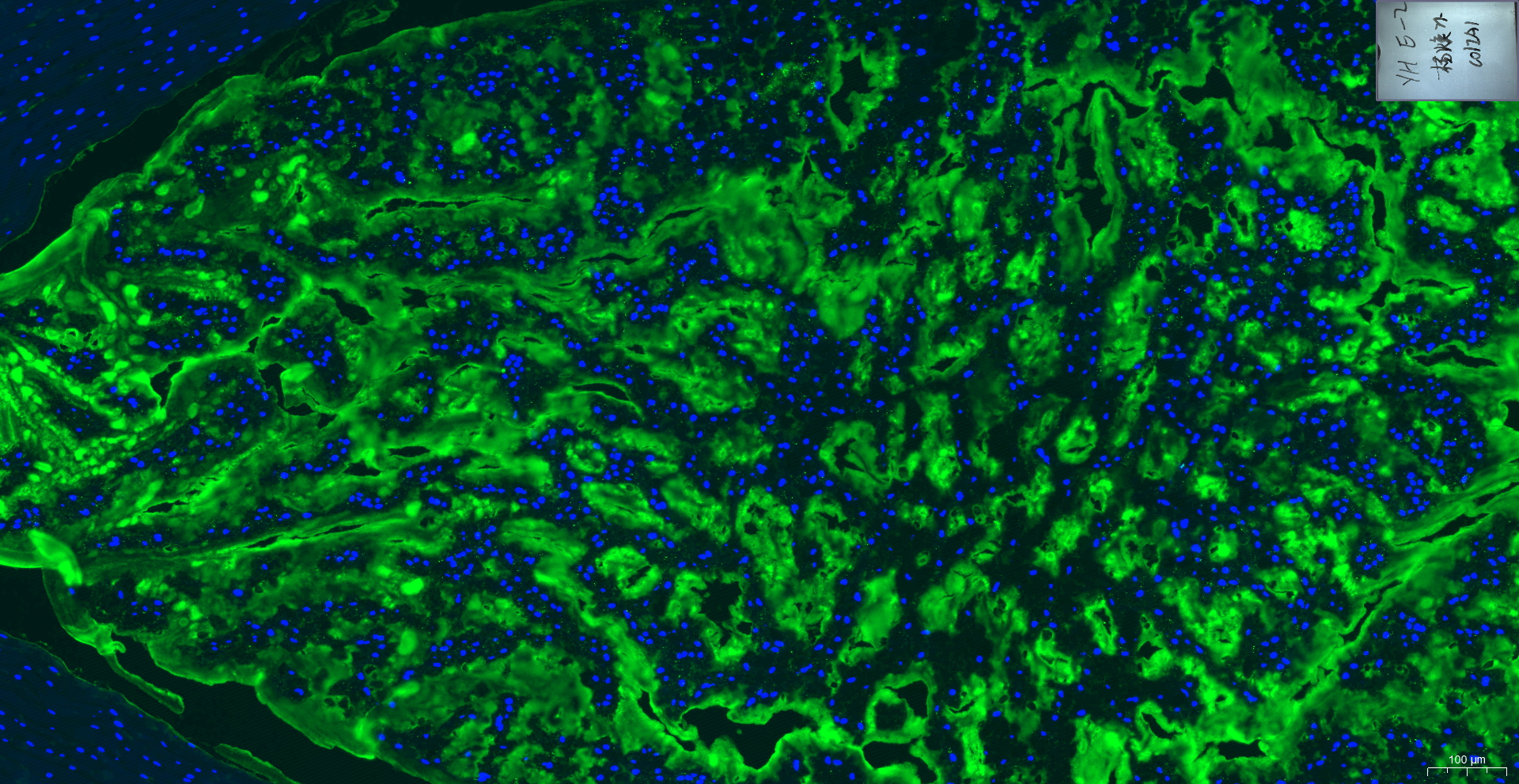

Supplement: Supplementary file 2 [file DataSheet1.ZIP › Raw Data/Figure 5 original data/E/Control_10.0x.jpg]

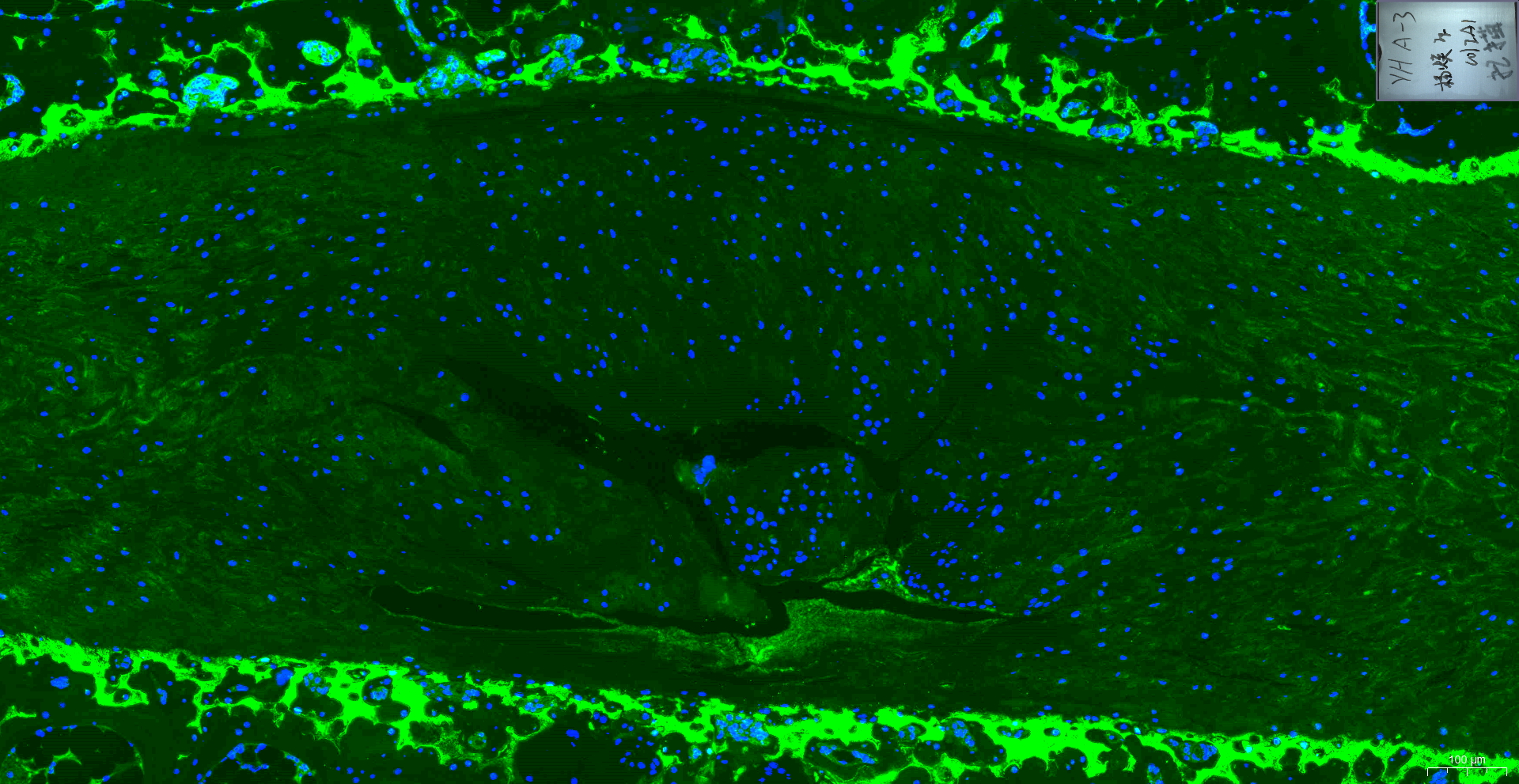

Supplement: Supplementary file 2 [file DataSheet1.ZIP › Raw Data/Figure 5 original data/E/EP 12.5uM.jpg]

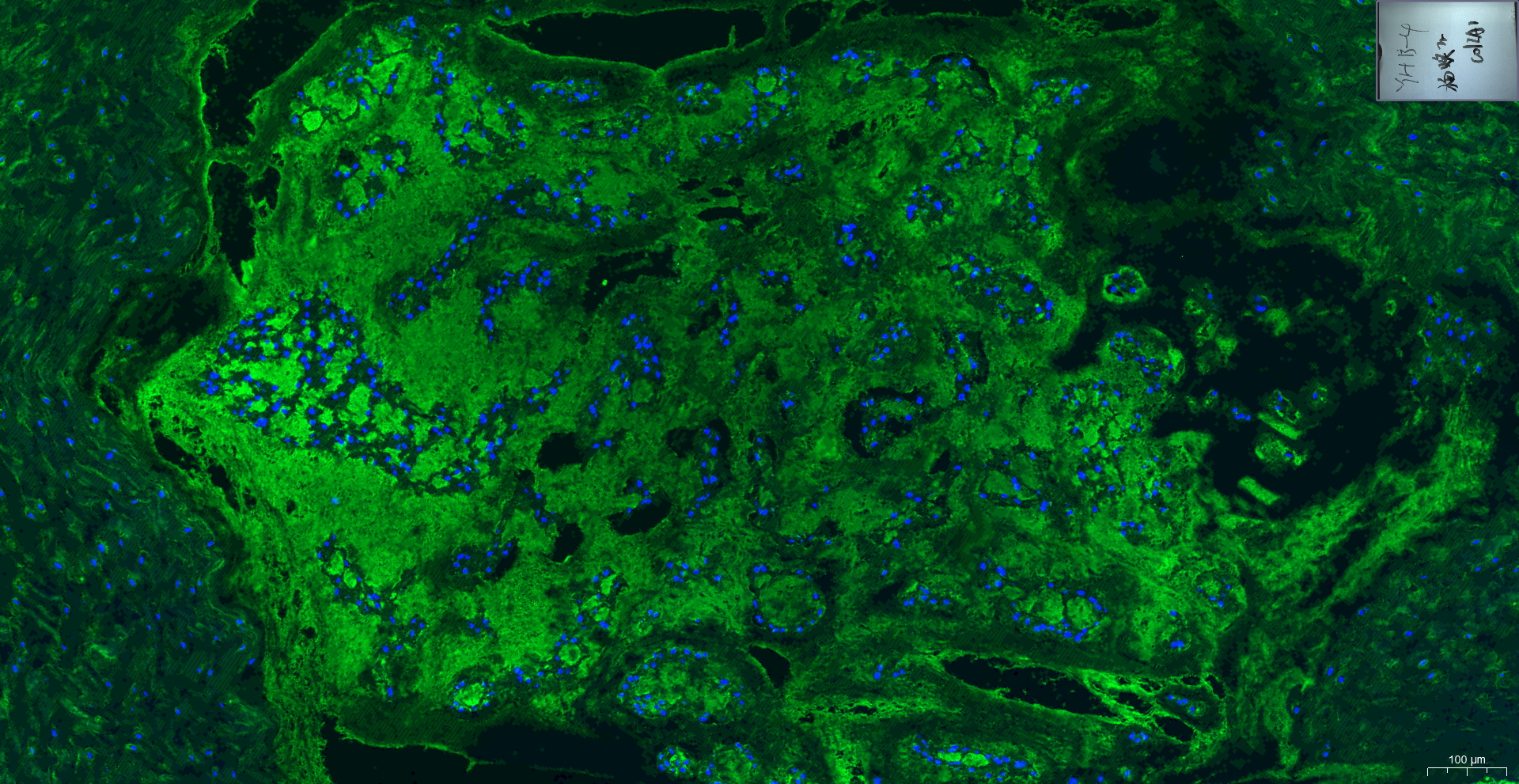

Supplement: Supplementary file 2 [file DataSheet1.ZIP › Raw Data/Figure 5 original data/E/EP 25uM.jpg]

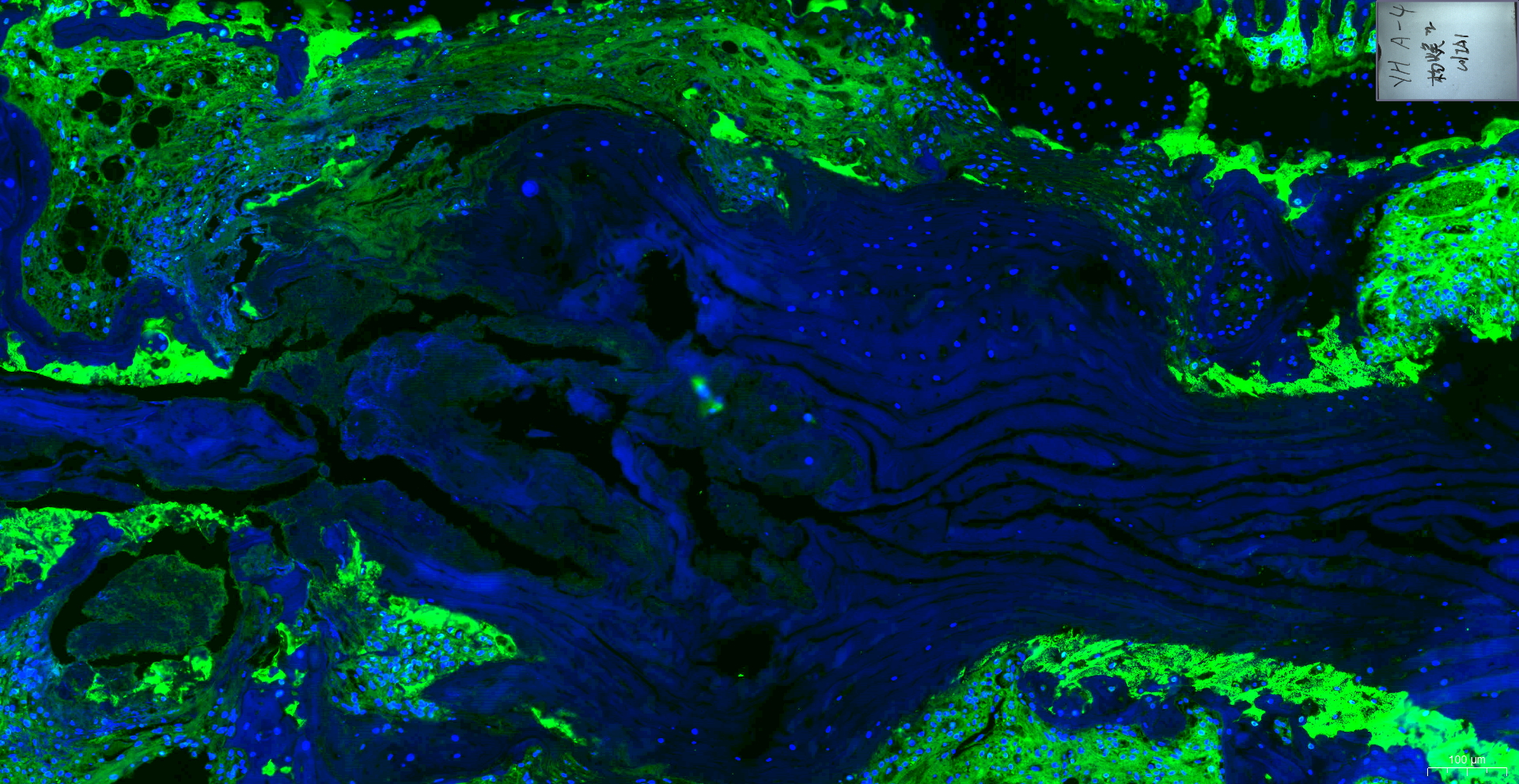

Supplement: Supplementary file 2 [file DataSheet1.ZIP › Raw Data/Figure 5 original data/E/PBS.jpg]
